# Synthesis of Polycyclic Ether-Benzopyrans and In Vitro Inhibitory Activity against *Leishmania tarentolae*

**DOI:** 10.3390/molecules25225461

**Published:** 2020-11-21

**Authors:** Sarita Singh, Jacob P. Grabowski, Shilpa Pohani, C. Fiore Apuzzo, David C. Platt, Marjorie A. Jones, T. Andrew Mitchell

**Affiliations:** Department of Chemistry, Illinois State University, Campus Box 4160, Normal, IL 61790-4160, USA; meet2sarita@gmail.com (S.S.); jpgrabo@ilstu.edu (J.P.G.); shilpa.pohani@gmail.com (S.P.); cfapuzz@ilstu.edu (C.F.A.); dcplatt@ilstu.edu (D.C.P.)

**Keywords:** oxidopyrylium, cycloaddition, Suzuki-Miyaura, benzopyran, oxabicyclo[3.2.1]-octane, Leishmania, secreted acid phosphatase, nitric oxide

## Abstract

Construction of a focused library of polycyclic ether-benzopyrans was undertaken in order to discover new therapeutic compounds that affect *Leishmania* growth and infectivity. This is especially of interest since there are few drug therapies for leishmaniasis that do not have serious drawbacks such high cost, side effects, and emerging drug resistance. The construction of these polycyclic ether-benzopyrans utilized an acetoxypyranone-alkene [5+2] cycloaddition and the Suzuki-Miyaura cross-coupling. The multi-gram quantity of the requisite aryl bromide was obtained followed by effective Pd-catalyzed coupling with boronic acid derivatives. Compounds were tested in vitro using the parasitic protozoan, *Leishmania tarentolae*. Effects of concentration, time, and exposure to light were evaluated. In addition, the effects on secreted acid phosphatase activity and nitric oxide production were investigated, since both have been implicated in parasite infectivity. The data presented herein are indicative of disruption of the *Leishmania tarentolae* and thus provide impetus for the development and testing of a more extensive library.

## 1. Introduction

**Figure 1:**
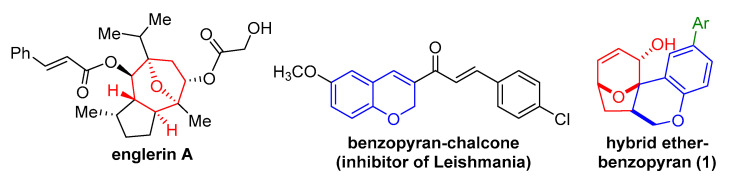
Hybrid polycyclic ether-benzopyran (**1**)**.**

According to the Centers for Disease Control and Prevention (CDC), leishmaniasis is a neglected tropical disease caused by parasitic protozoans in the genus *Leishmania* [1] that affects humans and animals in nearly 100 countries [2] with cases reported on every continent except Australia and Antarctica [3]. The exact number of leishmaniasis cases is not known but is reported to be between 1.5 and 2.0 million new cases per year with more than 350 million people at risk worldwide [4]. Although less common in the United States than many other parts of the western hemisphere [5], cases of cutaneous leishmaniasis have been documented in Oklahoma and Texas [6,7], raising concerns that this neglected disease will become a US public health issue sooner rather than later [8]. Leishmaniasis is caused by 20 different species of *Leishmania* and is spread by the bite of approximately 30 different species of phlebotomine sandflies [2]. Leishmaniasis is presented clinically in three different forms: cutaneous, visceral, or mucosal [2,9]. Although several treatments exist, they are not without liabilities [10,11]. For example, amphotericin B is cost-prohibitive, antimony compounds are toxic, and both therapies have significant side-effects. There are three main modes of therapy, including systemic parenteral, systemic oral, and local (i.e., topical application). Examples of systemic parenteral therapies include various drug formulations of pentavalent antimony (Sb^+5^) compounds. Systemic oral therapy may include the phosphocholine derivative miltefosine or a triazole-containing small molecule such as fluconazole. Examples of local therapies range from cryotherapy with liquid nitrogen to thermotherapy with localized current field radiofrequency heat, in addition to the intralesional administration of antimony-based compounds or topical application of paromomycin [12]. These treatments can be expensive and may result in harsh side effects with reports of drug resistance becoming a serious problem, so there is a critical need for new drugs to treat this neglected tropical disease [1,2]. Recently, a polycyclic ether-containing natural product, englerin A, was reported to be toxic in renal cancer cells and has become a very popular synthetic target (Figure 1) [13,14,15,16]. One hypothesis regarding the mechanism of action is a complex array of metabolic pathways involving PKCθ and HSF1, leading to an inhibition of glucose transporter (Glut1) activity [17]. In addition, Feng et al. reported that glucose transporters are critical for *Leishmania mexicana* viability and infectivity [18]. Furthermore, benzopyrans [19,20,21] are classified as privileged structures toward medicinal chemistry efforts [22] and benzopyran-chalcones have been shown to inhibit *Leishmania major* [23,24]. Thus, we hypothesized that hybrid oxabicyclo[3.2.1]-octane-benzopyrans **1** offered a unique scaffold to pursue inhibitory activity against the protozoan parasite *Leishmania tarentolae* [25]. This strain of *Leishmania* is generally regarded as non-pathogenic to humans, and it therefore serves as a model system in vitro [26]. We observed that these compounds negatively affect parasite growth and viability in vitro with mechanistic targets such as nitric oxide production and secreted acid phosphatase activity, both of which are proposed to be involved in parasite-host interactions [27,28]. We also included tests against mammalian glial cells, in culture, to evaluate toxicity for another cell type.

**Figure 2:**
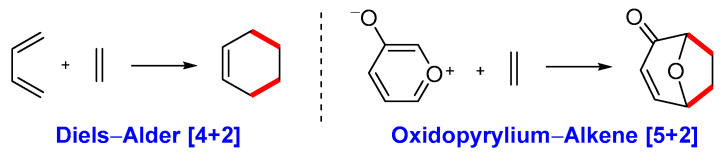
Diels–Alder [4+2] cycloaddition versus oxidopyrylium-alkene [5+2] cycloaddition.

In addition to the total synthesis of natural products [29,30,31,32,33,34,35,36], there is constant demand for the synthesis of unnatural heterocycles to access novel chemical space [37,38,39,40,41,42,43,44,45]. Although not authentic derivatives of secondary metabolites, these moieties resemble inherently diverse natural products [46,47,48,49,50,51,52]. Using natural products as metaphorical lead compounds, important heterocyclic scaffolds have been developed that would have otherwise remained undiscovered. Toward this end, cycloadditions provide an efficient means to synthesize valuable three-dimensional rings [53,54,55]. For example, Diels–Alder [4+2] cycloadditions are ubiquitous among organic reactions [56,57,58], while [5+2] cycloadditions are substantially less recognized [59,60]. However, polycyclic ethers are very important heterocyclic ring systems [61], and oxidopyrylium-based [5+2] cycloadditions (Figure 2) afford valuable oxabicyclo[3.2.1]-octanes [62,63,64] which are embedded within such natural products [65,66,67,68,69,70] as englerin A [13,14,15,16], platensimycin [71,72], cortistatins [73,74], and many others [75,76,77].

## 2. Results and Discussion

### 2.1. Previous Synthesis of Polycyclic Ether-Benzopyran

**Scheme 1:**
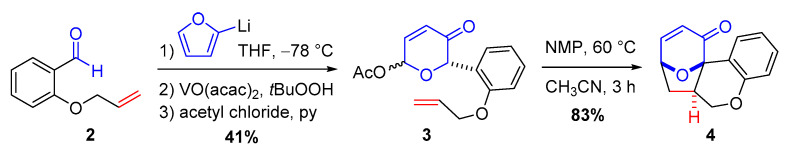
Previous synthesis of a polycyclic ether-benzopyran **3.**

As part of our research program directed toward [5+2] cycloadditions [78,79,80,81,82,83,84] we explored mechanistic pathways of acetoxypyranone-derived oxidopyrylium species en route to [5+2] cycloadditions [81] that led to the discovery of polycyclic ether-benzopyran **4** (Scheme 1). Commercially available aldehyde **2** was treated with 2-furyllithium to provide the desired alcohol (not shown) in quantitative yield. Achmatowicz rearrangement [85,86,87,88] followed by acylation afforded the acetoxypyranone **2** as a mixture of diastereomers in 41% yield over three steps. Finally, *N*-methylpyrrolidine (NMP)-mediated [5+2] cycloaddition [83] delivered the ether-benzopyran **4** in 83% yield.

### 2.2. Proposed Suzuki-Miyaura Pathway toward Construction of Ether-Benzopyran Analogues

**Scheme 2:**
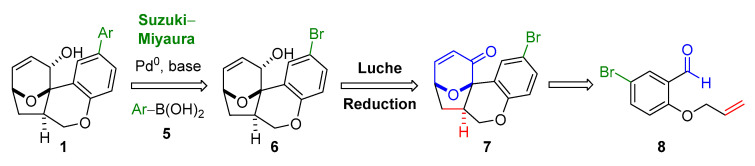
Proposed Suzuki-Miyaura pathway for the construction of ether-benzopyran analogues **1.**

Toward construction of a library of polycyclic ether-benzopyran analogues **1**, we envisioned the synthesis of aryl bromide **6** that would be suitable for Suzuki cross-coupling [89,90] with biologically relevant [91] boronic acid derivatives **5** (Scheme 2). The alcohol **6** would be accessible via Luche reduction [92] of enone **7**, which would arise from the key [5+2] cycloaddition. The cycloadduct **7** would be accessible by the aforementioned sequence (cf. Scheme 1) utilizing aryl bromide **8**, which is readily accessible upon the allylation of commercially available 5-bromo-salicyladehyde (not shown).

### 2.3. Construction of the Suzuki-Miyaura Aryl Bromide Precursor

**Scheme 3:**
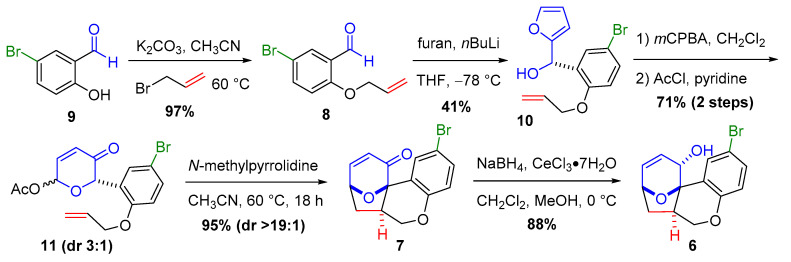
Synthesis of Suzuki-Miyaura cross-coupling partner **6.**

En route to cross-coupling partner **6**, commercially available 5-bromosalicylaldehyde **9** was heated in acetonitrile with allyl bromide and K_2_CO_3_ to give allyl ether **8** in 97% yield (Scheme 3). The treatment of furan with *n*BuLi in THF provided the 2-furyllithium, which underwent addition to aldehyde **8** to afford the alcohol **10**. Next, *m*CBPA-mediated Achmatowicz rearrangement [85,86,87,88] delivered the hydroxypyranone (not shown), which was acylated to give the acetoxypyranone **11** in 71% yield over two steps. NMP was utilized to promote the [5+2] cycloaddition [83] to afford the ketone **7** in 95% yield as a single diastereomer. Luche reduction [92] gave the allylic alcohol **6,** which was included to provide a hydrogen-bond donor and remove the reactive enone functionality [93].

### 2.4. Palladium Catalyzed Suzuki-Miyaura Cross-Coupling

Suzuki-Miyaura cross-coupling [89,90] is one of the most widely used reactions to construct C-C bonds and affords an effective delivery of relevant heterocycles [91]. Aryl bromide **6** provides the necessary functional group handle to react with boronic acid derivatives **5a**–**e** with catalytic palladium to deliver products **1a**–**e** (Table 1). In general, Pd(Ph_3_)_4_ was utilized as the catalyst with Cs_2_CO_3_ in a mixed solvent system of toluene, DME, and water to afford the desired cross-coupling products **1a**,**c**–**e** (entries 1, 3–5). However, in the case of 2-fluoropyridine-4-boronic acid **5b** (entry 2), Pd(dppf)Cl_2_ and K_2_CO_3_ in toluene:EtOH provided the desired product **1b** [94]. In all cases, the starting material **6** was consumed according to ^1^H NMR analyses of the crude reaction mixtures.

### 2.5. Biological Investigations

#### 2.5.1. Incubation Effects on L. tarentolae Growth

**Figure 3:**
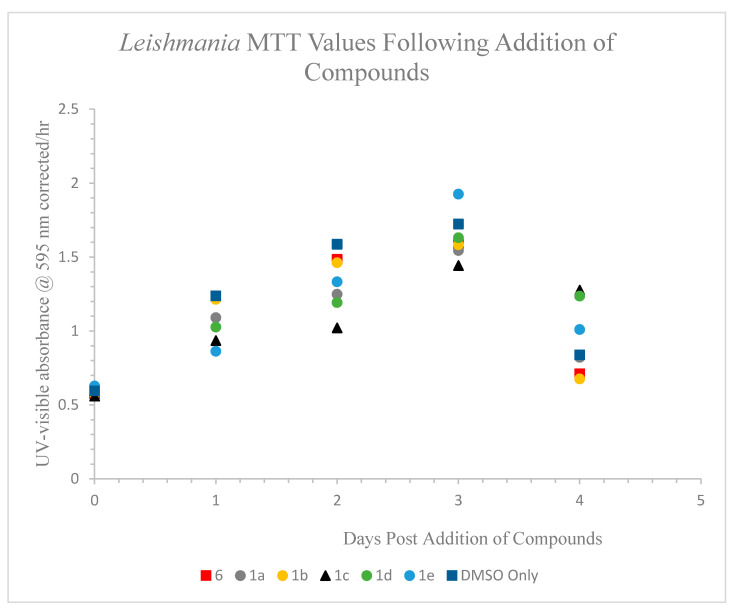
3-(4,5-dimethylthiazol-2-yl)-2,5-diphenyltetrazolium bromide (MTT) absorbance values from cells exposed to a single dose of 30 µM polycyclic ether-benzopyrans in DMSO were obtained at each interval (0 h and every 24 h subsequently). Values are the mean for *n* = 4 replicates and all SD were smaller than 2% of the mean. DMSO cells are considered to be the appropriate control.

No major differences were observed via light microscopy (data not shown) in the morphology or motility of *L. tarentolae* following 96-h incubations. The 3-(4,5-dimethylthiazol-2-yl)-2,5-diphenyltetrazolium bromide (MTT) viability assay [95] was used to evaluate cell viability with and without the test compounds. Initial MTT values were essentially the same, suggesting that the same number of viable cells were present in each of the separate flasks prior to the addition of the polycyclic ether-benzopyrans. Subsequent MTT values were altered in the presence of some of the test compounds in comparison to the DMSO control (Figure 3) and this is especially evident on day 2 post addition of the **1e** compound. On day 1 post addition of compounds **1a**,**1c**, **1d**, and **1e**, values were significantly different (*p*, 0.05) from DMSO control cells. One day 2, all additions resulted in significant differences relative to DMSO control cells. On day 3, all additions resulted in significant differences except compound **1d**. On day 4, all additions resulted in significant differences except compound **1a**. It is of interest that compound 1e has an apparent early inhibitory effect on cells, relative to control cells, but that, with time, the compound is not stable, so the inhibition is relieved, and the cells recover.

#### 2.5.2. Test Compound Inhibition and Apparent Recovery by Leishmania after Transfer to New Medium

**Figure 4:**
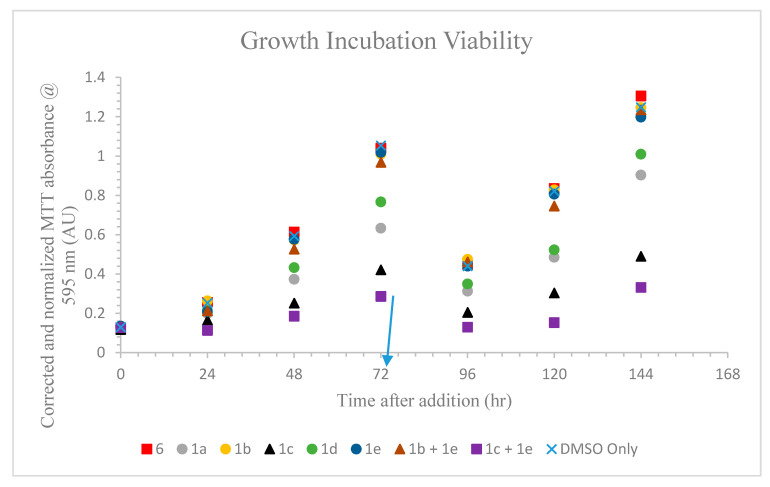
MTT values at 24 h intervals post addition of a single dose of a polycyclic ether-benzopyran. The compounds (30 µM) were added directly after first reading. Arrow indicates time that cells were given fresh medium with no added compound (recovery phase). Values are the mean values from *n* = 4 replicates.

Inhibition of *L. tarentolae* growth was seen by several compounds (Figure 4) with the most effective inhibitory compounds being compound **1c** and co-incubation of compounds **1c** + **1e**. Slopes obtained for the original growth period and the secondary recovery phase indicate some level of recovering capabilities after exposure to different compounds (Table 2), since a greater ratio of the slope corresponds to an increased ability to recover after compound exposure. For all days post addition of compounds **1a**, **1c**, **1d**, and co-incubation with **1c** + **1e**, there was a statistically significant change relative to control cells (*p* < 0.05).

Significant differences were observed in the differential growth patterns of experimental cultures compared to DMSO control. Complexes appear to be affecting various pathways involved in cell maturation and division. Growth curve plots show severe growth inhibition in the presence of multiple compounds. Although indole **1c** shows the highest inhibitory activity, this compound also reveals synergistic activity in the presence of pyrimidine **1e**. The difference in recovery is minimal between each sample when comparing the ratio of the slopes. No condition appeared to bring the cellular viability to 0. However, this was a one-dose study (addition at 0 h) and it is likely that a multidose study would further affect the cellular viability of the parasite.

#### 2.5.3. Incubation Effects of Secreted Acid Phosphatase (SAP) Activity

**Figure 5:**
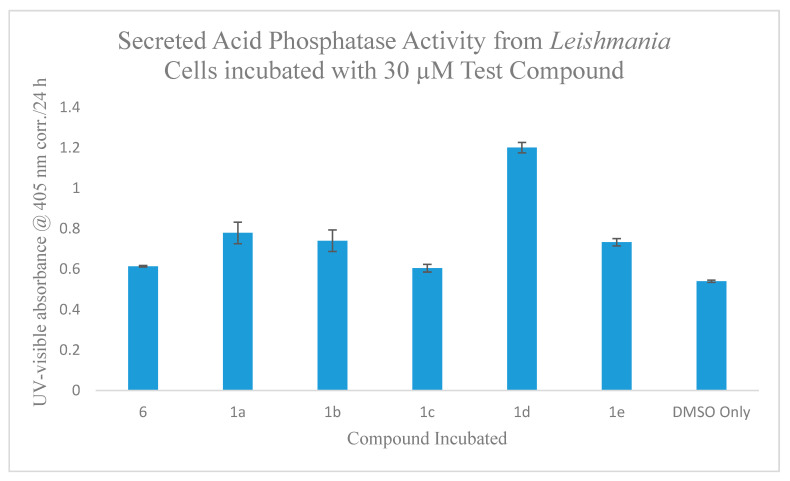
Absorbance (A 405 nm) after 24 h incubation with *p*NPP. Values are mean ± SD for *n* = 4 replicates.

**Figure 6:**
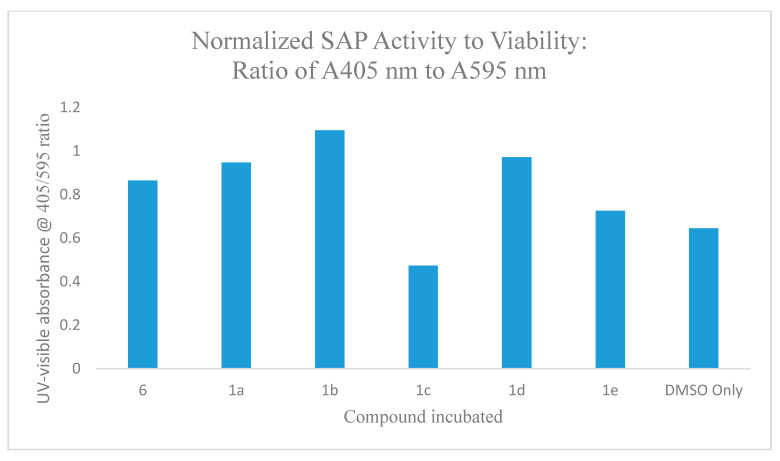
Normalized secreted acid phosphatase (SAP) assay absorbance to MTT absorbance per condition.

Secreted acid phosphatase (SAP) activity on the fourth day (96 h post-addition) showed a marked difference between control and experimental conditions (Figure 5). Normalizing the absorbance obtained for SAP activity to the day 4 MTT absorbance suggests that SAP activity is directly or indirectly affected in the presence of the test compounds (Figure 6). For all compounds tested, there was a significant increase (*p* < 0.05) in secreted acid phosphatase activity relative to DMSO control cells.

#### 2.5.4. Standard SAP Direct Enzymic Effect Assay

**Figure 7:**
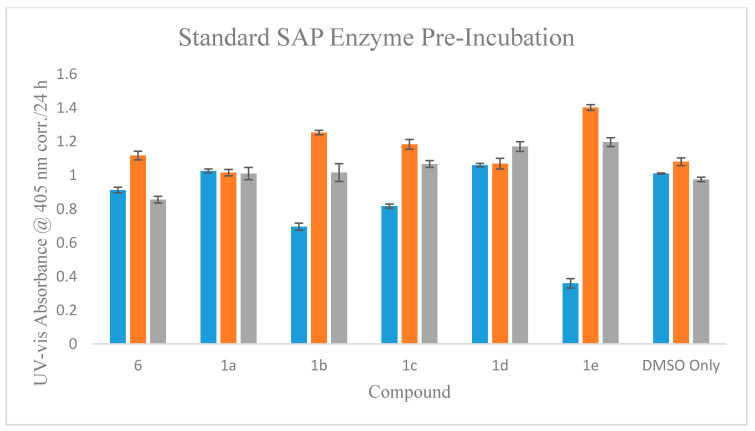
Absorbance values (A 405 nm) resulting from differing pre-incubation with polycyclic ether-benzopyrans. Values are the mean ± SD for *n* = 4 replicates. Blue: 15 min; orange: 18 h; gray: 46 h.

A 15-min pre-incubation period before substrate addition showed a decrease in SAP activity with compounds **1b** and **1e** being the most effective (Figure 7). The 18-h and 46-h pre-incubation periods showed little difference to control in terms of SAP activity, suggesting that enzymatic inhibition may have occurred during the 15-min pre-incubation period, resulting in a lower enzyme activity and less product formed. However, with time, the enzyme activity appears to recover, suggesting that the test compounds may not be stable under these incubation conditions.

#### 2.5.5. Standard Leishmania SAP Enzyme Kinetic Assay

A 15-min pre-incubation period was allotted for the enzyme to interact with each test compound before starting the enzymatic reaction with the addition of substrate. V vs. [S] curves between conditions show no difference in enzyme activity depending on either test compound present or substrate concentration. Lineweaver-Burk plots for each condition yield very similar K_M_ and V_max_ values, suggesting no direct modulation as either an activator or inhibitor of *Leishmania* SAP activity (Table 3).

In all cases (cf. Figure 5), the detectable SAP activity was similar to or larger than the DMSO control cells, suggesting some effect on either the cells’ ability to synthesize or release this important parasite enzyme. It was of interest that incubation with compound **1d** resulted in the highest value of detectable SAP activity. When normalizing the absorbance obtained for SAP activity to the day 4 MTT absorbance, it appears that the SAP activity is directly or indirectly affected in the presence of the test compounds (Figure 6). Comparable values for both K_M_ and V_max_ for all conditions (Table 3) suggests no direct enzymic effect of activation or inhibition on SAP by **6**, **1c**, and **1d**. These data suggest that some test compounds affect the Leishmania cells’ ability to synthesize or release the secreted acid phosphatase (SAP) enzyme, but do not directly affect the enzyme activity.

#### 2.5.6. Detection of Leishmania Nitric Oxide Using a Specific Fluorescent Probe, DAF-FM

Aliquots (equal volumes from each condition) of cells were centrifuged, resuspended and incubated, washed, and plated for microscopy. All compounds showed a modest decrease in NO production relative to the 1% DMSO control (Table 4). However, **1b**, **1e**, and co-incubation with **1b** + **1e** show a more substantial decrease in NO levels than the other compounds. These trends are shown in the microscope images (Figures S15–S20) at 40x magnification in which green indicates cells responding to the probe for nitric oxide. Note that there were no observable differences in cell morphology when comparing control (with DMSO) to experimental cells. This is of interest since the production of nitric oxide by *Leishmania* has been proposed as one mechanism by which these parasites protect themselves from oxidative stress [27]. Then a reduction in nitric oxide production may render these parasites less infective to host cells. Further studies should be conducted using standard inducible nitric oxide synthase (iNOS) to test if either of these two compounds acts as a direct enzyme inhibitor.

#### 2.5.7. Cell Toxicity Screening: C6 Glial Cells

No acute toxicity was seen in glial cells following a 2-h incubation with the various test compounds relative to DMSO control (Table 5). In contrast to the viability assays with *L tarentolae*, our investigations of all test compounds were not found to be acutely toxic to glial cells in vitro. This suggests that these compounds may not be as hazardous to mammalian cells. Short term incubation, with other compounds, has been shown previously to elicit acute cytotoxicity in glial cells [96]. A therapeutic use of these compounds targeted toward inhibiting *Leishmania* based infection should be further investigated.

#### 2.5.8. ED-50 (Effective Dose) Study on Leishmania

The toxicity assay was replicative of earlier incubation results, suggesting no toxicity from **6**, and a cytotoxic effect from **1c** and **1e**. The formation of a trendline was used on the linear portion for both **1c** and **1e** to estimate an ED-50 concentration for both days after addition (Table 6). Compound **6** had no apparent effect on the cell viability at levels up to 200 µM.

**Figure 8:**
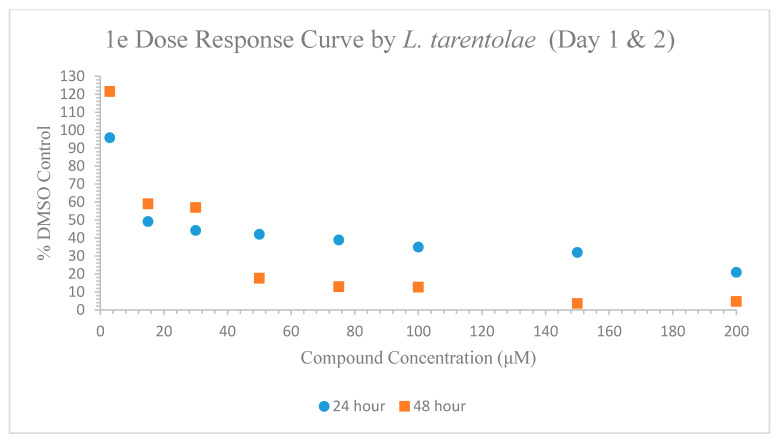
The dose-response curve at 24 and 48 h after a single addition of test compound **1e** at concentrations ranging from 4.5 to 200 µM.

Compounds **1c** and **1e** clearly show some level of toxicity to *L. tarentolae*, whereas **6** does not. The second day MTT measurement of **1c** incubated *L. tarentolae* decreased from the first day suggesting that the *L. tarentolae* may be unable to recover from exposure, especially at higher concentrations. This ability to recover is seen at lower (less than 40 µM) but not higher concentrations with **1e**, as shown in Figure 8.

#### 2.5.9. Photosensitivity study on Leishmania

No difference in light exposure sensitivity was seen immediately after uptake period and light exposure between experimental and control cultures (Table 7). However, by 24 h, cells with test compound **1c** exhibited a reduction in cell viability but independent of light treatment. In contrast, **1e** or **6** treated cells exhibited no toxicity throughout the entire trial under any light condition. In general, *L. tarentolae* appeared to be resistant to exposure to both UV and fluorescent light under these experimental conditions. Compound **1e** MTT levels are in line with DMSO control, which was unexpected, as all other studies showed some level of toxicity. One possible explanation is that this molecule is not as stable as the others and thus stock preparations undergoing multiple freeze-thaw cycles in DMSO from −20 °C to tepid water rendered this inactive. Compound **1c** continued to exhibit the same toxic effect to *L. tarentolae*, as it was not dependent on the light exposure received implying that light exposure has no effect on uptake and/or toxicity of **1c**.

## 3. Conclusions

Due to the pressing need for new small molecules to treat Leishmaniasis, hybrid polycyclic ether-benzopyrans were constructed in order to combine the potential glucose transporter inhibition with the privileged benzopyran structure. The construction of this new scaffold was undertaken by utilizing the complexity-generating [5+2] cycloaddition in conjunction with the Suzuki-Miyaura cross-coupling to deliver biologically relevant heterocycles. *Leishmania tarentolae* growth was shown to be affected in the presence of these organic molecules to differing extents with no noticeable change in cell morphology, motility, or clumping. Although the biochemical mechanisms are not fully understood, two different enzymes were evaluated in this study, namely secreted acid phosphatase and (indirectly) nitric oxide synthase. SAP secretion or transcriptional regulation may be affected by the presence of these polycyclic ether-benzopyrans, since we found that there is differing MTT normalized SAP readings from in vitro incubation. However, none of the compounds tested appear to have a direct, modulatory effect on SAP as an activator or inhibitor. The reduction in detectable nitric oxide by some of the test compounds was substantial and may reduce the ability of these cells to detoxify reactive oxygen species of host cells. Acute toxicity does not seem to occur in cultured glial cells indicating a differential effect relative to cell type. Polycyclic ether-benzopyrans **1c** and **1e** appear to have the most inhibitory effects on *Leishmania tarentolae* viability through a concentration and time-dependent manner. Exposure to different wavelengths of light does not seem to affect toxicity levels of these two complexes. Under some conditions, the compounds do not appear to be stable with time, suggesting that potential therapy will involve multiple dosing to bring about successful reduction of the parasitic load. Future work will focus on evaluation of the mechanism of action especially involving the GLUT transporter by which these polycyclic ether-benzopyrans affect *Leishmania* in vitro.

## 4. Materials and Methods

### 4.1. General Methods—Organic Synthesis

All reactions were performed under Ar atmosphere in oven-dried or flame-dried glassware unless otherwise noted. Diethyl ether (Et_2_O) was dried over pressed Na metal. All other commercially available anhydrous solvents and reagents were used as received. Sodium hydride (NaH) was a 60% dispersion in mineral oil. Thin layer chromatography was performed with glass or aluminum plates (silica gel F_254_, Art 5715, 0.25 mm), visualized by fluorescence quenching under UV light, and stained with potassium permanganate. Flash column chromatography (FCC) was performed with silica gel 60A 40–63 µm (200–400 mesh). Mass spectral data were acquired using positive mode Electrospray Ionization (ESI+) and a high-resolution Time of Flight (TOF) mass spectrometer (ThermoFisher Scientific, Waltham, MA, USA). ^1^H NMR spectra were acquired at 400 or 500 MHz and ^13^C{^1^H} NMR spectra were acquired at 100 MHz or 125 MHz as noted (Bruker, Billerica, MA, USA). ^1^H and ^13^C{^1^H} NMR chemical shifts are reported in ppm (δ) relative to the residual solvent peaks. ^1^H NMR coupling constants (*J*) are reported in Hertz (Hz), and multiplicities are indicated as follows: s (singlet), br. s (broad singlet), d (doublet), t (triplet), q (quartet), quint (quintet), sext (sextet), sept (septet), dd (doublet of doublets), ddd (doublet of doublet of doublets), dt (doublet of triplets), td (triplet of doublets), ddt (doublet of doublet of triplets), dq (doublet of quartets), qq (quartet of quartets), ovlp (overlapping), m (multiplet), app. (apparent). Based on intensity in the ^13^C{^1^H} spectra, both magnetic and chemical shift equivalent peaks are noted in parentheses. Compounds **6** and **1a-1e** were solubilized with DMSO to 30 mM for stock concentration (stored at −20 °C).

### 4.2. Preparation of Aryl Bromide 6

**Aryl bromide 6:** To a solution of phenol **9** (10.0 g, 49.7 mmol, 1.0 equiv) in CH_3_CN (100 mL) was added K_2_CO_3_ (10.30 g, 74.58 mmol, 1.5 equiv). Then allyl bromide was delivered to this solution dropwise via syringe. The reaction flask was fitted with an air reflux condenser and stirred for 2.5 h at 60 °C. Then, the reaction was cooled to room temperature and quenched by the addition of water (100 mL). Extraction with ethyl acetate (2 × 100 mL) afforded an organic layer that was washed with 1M NaOH (100 mL) and brine (100 mL), dried with MgSO_4_, filtered, and concentrated. Crude allyl ether **8** was filtered through a plug of silica with diethyl ether and carried on without further purification (11.72 g, 48.5 mmol, 97%). To a solution of furan (1.40 mL, 19.9 mmol, 1.0 equiv) in THF (15 mL) cooled to −78 °C was added *n*BuLi (9 mL, 23.8 mmol, 1.2 equiv) dropwise via syringe. The reaction was stirred for 1.5-h at –78 °C, and then warmed to 0 °C for 30 min. Upon cooling back to –78 °C, aldehyde **8** (9.6 g, 39.8 mmol, 2.0 equiv) in THF (45 mL) was added dropwise via syringe. The reaction was stirred for 1-h at –78 °C followed by 30 min at 23 °C. The reaction was quenched by the slow addition of water (100 mL) at 0 °C and extracted with Et_2_O (300 mL). The combined organic layer was washed with sat. aq. NaCl (100 mL), dried with MgSO_4_, filtered, and concentrated. Purification by FCC (hexanes:ethyl acetate 90:10) afforded furfuryl alcohol **10** as a yellow solid (2.5 g, 8.1 mmol, 41%). To a solution of furfuryl alcohol **10** (8.0 g, 26.0 mmol, 1.0 equiv) in CH_2_Cl_2_ (200 mL) was added *m*CPBA (8.32 g, 33.75 mmol, 1.3 equiv) at 0 °C. Then, the reaction mixture was stirred for 5 h at 23 °C and quenched by the addition of sat. aq. Na_2_S_2_O_3_ (50 mL), sat. aq. NaHCO_3_ (50 mL), and 10% aq. Na_2_CO_3_ (50 mL). The resulting mixture was extracted with CH_2_Cl_2_ (200 mL), dried with Na_2_SO_4_, filtered, and concentrated. Purification by FCC (CH_2_Cl_2_:Et_2_O 95:5) afforded hydroxypyranone (not shown) as a white solid (6.09 g, 18.7 mmol, 72%, dr 3:1). To a solution of hydroxypyranone (6.09 g, 18.7 mmol, 1.0 equiv) in CH_2_Cl_2_ (150 mL) was added pyridine (3.77 mL, 46.8 mmol, 2.5 equiv) and AcCl (2.0 mL, 28.1 mmol, 1.5 equiv) at 0 °C. The reaction was stirred for 2 h, quenched with sat. aq. NaCl (100 mL), and then separated. The aqueous layer was extracted with CH_2_Cl_2_ (200 mL), and the organic layer was dried with Na_2_SO_4_, filtered, and concentrated. Purification by FCC (CH_2_Cl_2_:Et_2_O 95:5) afforded acetoxypyranone **11** as a pale yellow solid (6.74 g, 18.4 mmol, 98%). To a solution of acetoxypyranone **11** (6.7 g, 18.4 mmol, 1.0 equiv) in CH_3_CN (120 mL) was added NMP (7.64 mL, 73.4 mmol, 4.0 equiv). The reaction was heated to 60 °C for 18 h and concentrated. Purification by FCC (CH_2_Cl_2_:Et_2_O 95:5) afforded enone **7** as a white solid (5.38 g, 17.5 mmol, 95%, dr > 19:1): *R*_f_ = 0.74 (CH_2_Cl_2_:Et_2_O 85:15); mp 184 °C; ^1^H NMR (500 MHz, CDCl_3_) δ 7.41 (dd, *J =* 9.9, 4.6 Hz, 1H), 7.39–7.35 (m, 2H), 6.90-6.86 (m, 1H), 6.17 (d, *J =* 9.9 Hz, 1H), 4.88 (app dd, *J =* 7.1, 4.6 Hz, 1H), 4.42 (dd, *J =* 10.9, 5.8 Hz, 1H), 3.45 (dd, *J =* 12.1, 10.9 Hz, 1H), 2.58–2.51 (m, 1H), 2.24 (ddd, *J =* 12.3, 8.4, 0.6 Hz, 1H), 1.86 (ddd, *J =* 12.3, 7.1, 4.6 Hz, 1H); ^13^C{^1^H} NMR (100 MHz, CDCl_3_) δ 196.0, 155.6, 153.6, 133.3, 133.0, 127.2, 122.3, 119.3, 113.9, 85.1, 72.5, 68.1, 36.4, 33.6; ESI-HRMS calculated for C_14_H_11_BrO_3_Na [M + Na]^+^ 328.9789 (Br^79^) and 330.9769 (Br^81^), found 328.9784 and 330.9760. To a solution of enone **7** (5.38 g, 17.5 mmol, 1.0 equiv) in CH_2_Cl_2_:MeOH (30 mL:120 mL) was added CeCl_3•_7H_2_O (13.06 g, 35.0 mmol, 2.0 equiv) at 23 °C. Upon stirring for 30 min, the reaction was cooled to 0 °C and NaBH_4_ (500 mg, 13.14 mmol, 0.75 equiv) was added slowly. Upon stirring for 30 min at 0 °C, a second portion of NaBH_4_ (500 mg, 13.14 mmol, 0.75 equiv) was added slowly. The reaction was stirred at 0 °C for 1 h and was quenched via slow addition of sat. aq. NaCl (100 mL). This biphasic solution was diluted with Et_2_O (200 mL) and separated. The combined organic layer was dried with Na_2_SO_4_, filtered, and concentrated. Purification by FCC (CH_2_Cl_2_:Et_2_O 95:5) afforded aryl bromide **6** as a white solid (4.79 g, 15.5 mmol, 88%): *R*_f_ = 0.63 (CH_2_Cl_2_:Et_2_O 85:15); mp 133–134 °C; ^1^H NMR (500 MHz, CDCl_3_) ^1^H NMR (500 MHz, CDCl_3_) δ 7.69 (d, *J =* 2.4 Hz, 1H), 7.32 (dd, *J =* 8.7, 2.4 Hz, 1H), 6.86 (d, *J =* 8.7 Hz, 1H), 6.09 (ddd, *J =* 9.8, 4.1, 1.8 Hz, 1H), 5.65 (dd, *J =* 9.8, 2.1 Hz, 1H), 4.77–4.73 (m, 1H), 4.55 (dd, *J =* 6.0, 4.1 Hz, 1H), 4.37 (dd, *J =* 9.9, 5.4 Hz, 1H), 3.34 (dd, *J =* 12.3, 9.9 Hz, 1H), 3.34–3.24 (m, 1H), 2.18 (dd, *J =* 11.6, 7.8 Hz, 1H), 1.62–1.57 (m, 1H), 1.38 (d, *J =* 4.7 Hz, 1H); ^13^C{^1^H} NMR (125 MHz, CDCl_3_) δ 156.4, 133.6, 132.3, 131.0, 127.7, 127.1, 119.3, 114.5, 80.4, 74.7, 73.5, 69.9, 36.5, 34.4; ESI-HRMS calculated for C_14_H_13_BrO_3_Na [M + Na]^+^ 330.9946 (Br^79^) and 332.9925 (Br^81^), found 330.9945 and 332.9945.

### 4.3. Preparation of Biaryls 1**a**–**e**

**Suzuki-Miyaura Cross-Coupling General Procedure:** To a solution of aryl bromide **6** in either toluene:EtOH (3:1 ratio) or toluene:DME:H_2_O (1:1.3:2.9 ratio) was added boronic acid or derivative **5a**–**e** (1.2–1.5 equiv). Either aq. 2 M K_2_CO_3_ (6.0 equiv) or Cs_2_CO_3_ (3.5 equiv) was added followed by purging of the reaction mixture with Ar for 10 min. Then, catalytic Pd(dppf)Cl_2_ (30 mol%) or Pd(PPh_3_)_4_ (5–10 mol%) was added with additional purging with Ar for 10 min before allowing the reaction to stir at 85–110 °C for 12–16 h. Then, reactions were allowed to cool to room temperature, concentrated, and extracted with CH_2_Cl_2_ (50 mL). The organic layer was dried with Na_2_SO_4_, filtered, concentrated, and purified via FCC to afford cross-coupling products **1a**–**e**.

**4-Methoxyphenyl Biaryl 1a:** General procedure A was followed with aryl bromide **6** (50 mg, 0.16 mmol, 1.0 equiv), toluene:DME:H_2_O (0.7 mL:0.9 mL:2.0mL), potassium-4-methoxyphenyl-trifluoroborate **5a** (46 mg, 0.19 mmol, 1.2 equiv), Cs_2_CO_3_ (183 mg, 0.56 mmol, 3.5 equiv), and Pd(PPh_3_)_4_ (9 mg, 0.008 mmol, 0.05 equiv) for 16 h at 85 °C. Purification by FCC (hexanes:EtOAc 80:20) delivered 4-methoxyphenyl biaryl **1a** as a pale yellow solid (38 mg, 0.11 mmol, 69%): *R*_f_ = 0.47 (hexanes:EtOAc 70:30); mp 72–73 °C; ^1^H NMR (400 MHz, CDCl_3_) δ 7.75 (d, *J =* 2.3 Hz, 1H), 7.50 (app d, *J =* 8.8 Hz, 1H), 7.43 (dd, *J =* 8.4, 2.3 Hz, 1H), 7.02 (d, *J =* 8.4 Hz, 1H), 6.95 (app d, *J =* 8.8 Hz, 1H), 6.12 (ddd, *J =* 9.8, 4.1, 1.8 Hz, 1H), 5.68 (dd, *J =* 9.8, 2.1 Hz, 1H), 4.85 (br s, 1H), 4.59 (dd, *J =* 6.0, 4.1 Hz, 1H), 4.41 (dd, *J =* 9.8, 5.5 Hz, 1H), 3.85 (s, 3H), 3.41 (dd, *J =* 12.3, 9.8 Hz, 1H), 3.44-3.30 (m, 1H), 2.21 (dd, *J =* 11.7, 7.9 Hz, 1H), 1.67-1.60 (m, 1H), 1.42 (br s, 1H); ^13^C{^1^H} NMR (100 MHz, CDCl_3_) δ 159.0, 156.4, 135.2, 133.7, 133.2, 127.9(2), 127.8, 127.7, 126.2, 124.9, 117.7, 114.3(2), 80.8, 74.9, 73.5, 69.9, 55.5, 36.7, 34.6; ESI-HRMS calculated for C_21_H_20_O_4_Na [M + Na]^+^ 359.1259, found 359.1243.

**2-Fluoropyridyl Biaryl 1b:** General procedure A was followed with aryl bromide **6** (50 mg, 0.16 mmol, 1.0 equiv), toluene:EtOH (3 mL:1 mL), 2-fluoropyridine-4-boronic acid **5b** (34 mg, 0.24 mmol, 1.5 equiv), aq. K_2_CO_3_ (0.4 mL, 0.98 mmol, 6.0 equiv) and Pd(dppf)Cl_2_ (36 mg, 0.049 mmol, 0.30 equiv) for 16 h at 110 °C. Purification by FCC (hexanes:EtOAc 80:20 to 60:40) delivered 2-fluoropyridyl biaryl **1b** as a white solid (20 mg, 0.06 mmol, 38%): *R*_f_ = 0.28 (hexanes:EtOAc 70:30); mp 186–187 °C; ^1^H NMR (500 MHz, CDCl_3_) δ 8.22 (app d, *J =* 5.3 Hz, 1H), 7.87 (app d, *J =* 2.3 Hz, 1H), 7.53 (app dd, *J =* 8.5, 2.3 Hz, 1H), 7.38 (app ddd, *J =* 5.3, 2.0, 1.5 Hz, 1H), 7.11–7.10 (m, 1H), 7.10 (app d, *J =* 8.5 Hz, 1H), 6.14 (ddd, *J =* 9.8, 4.1, 1.8 Hz, 1H), 5.68 (dd, *J =* 9.9, 2.1 Hz, 1H), 4.84–4.81 (m, 1H), 4.60 (dd, *J =* 6.2, 4.1 Hz, 1H), 4.45 (dd, *J =* 9.8, 5.4 Hz, 1H), 3.41 (dd, *J =* 12.4, 9.8 Hz, 1H), 3.41–3.31 (m, 1H), 2.23 (dd, *J =* 11.6, 7.8 Hz, 1H), 1.67–1.61 (m, 1H), 1.45 (d, *J =* 4.8 Hz, 1H); ^13^C{^1^H} NMR (125 MHz, CDCl_3_) δ 165.7, 163.8, 158.8, 153.4 (*J =* 8.3 Hz), 148.0 (*J =* 15.8 Hz), 133.8, 131.1 (*J =* 3.2 Hz), 127.9 (*J =* 14.6 Hz), 127.1, 125.9, 119.2 (*J =* 3.6 Hz), 118.5, 106.6 (*J =* 38.0 Hz), 80.6, 75.0, 73.6, 70.1, 36.7, 34.6; ESI-HRMS calculated for C_19_H_17_NO_3_F [M + H]^+^ 326.1192, found 326.1179.

**6-Indole Biaryl 1c:** General procedure A was followed with aryl bromide **6** (50 mg, 0.16 mmol, 1.0 equiv), toluene:DME:H_2_O (0.7 mL:0.9 mL:2.0mL), 6-indoleboronic acid **5c** (39 mg, 0.24 mmol, 1.5 equiv), Cs_2_CO_3_ (78 mg, 0.56 mmol, 3.5 equiv) and Pd(PPh_3_)_4_ (9 mg, 0.008 mmol, 0.05 equiv) for 16 h at 85 °C. Purification by FCC (CH_2_Cl_2_:EtOAc 90:10) delivered 6-indole biaryl **1c** as a pale yellow-white solid (11 mg, 0.03 mmol, 19%): *R*_f_ = 0.67 (CH_2_Cl_2_:EtOAc 85:15); mp 70–73 °C; ^1^H NMR (500 MHz, CDCl_3_) δ 8.17 (br s, 1H), 7.86 (d, *J =* 2.3 Hz, 1H), 7.67 (d, *J =* 8.2 Hz, 1H), 7.57–7.55 (m, 1H), 7.54 (dd, *J =* 8.5, 2.3 Hz, 1H), 7.37 (dd, *J =* 8.2, 1.6 Hz, 1H), 7.22 (dd, *J =* 3.1, 2.3 Hz, 1H), 7.04 (d, *J =* 8.5 Hz, 1H), 6.56 (app ddd, *J =* 3.1, 2.0, 0.9 Hz, 1H), 6.12 (ddd, *J =* 9.9, 4.1, 1.8 Hz, 1H), 5.68 (dd, *J =* 9.9, 2.1 Hz, 1H), 4.87 (br s, 1H), 4.59 (dd, *J =* 6.0, 4.1 Hz, 1H), 4.41 (dd, *J =* 10.0, 5.8 Hz, 1H), 3.43 (dd, *J =* 12.3, 10.0 Hz, 1H), 3.39-3.29 (m, 1H), 2.22 (dd, *J =* 11.6, 8.0 Hz, 1H), 1.68–1.60 (m, 1H), 1.49 (d, *J =* 4.4 Hz, 1H); ^13^C{^1^H} NMR (125 MHz, CDCl_3_) δ 156.4, 136.7, 136.6, 135.0, 133.8, 128.4, 127.8, 127.2, 126.8, 124.85, 124.77, 121.0, 119.7, 117.7, 109.3, 102.7, 80.9, 75.0, 73.6, 70.0, 36.7, 34.7; ESI-HRMS calculated for C_22_H_19_NO_3_Na [M + Na]^+^ 368.1263, found 368.1250.

**Benzothiazole Biaryl 1d:** General procedure A was followed with aryl bromide **6** (50 mg, 0.16 mmol, 1.0 equiv), toluene:DME:H_2_O (0.7 mL:0.9 mL:2.0mL), benzothiazole-5-boronic acid pinacol ester **5d** (63 mg, 0.24 mmol, 1.5 equiv), Cs_2_CO_3_ (78 mg, 0.56 mmol, 3.5 equiv) and Pd(PPh_3_)_4_ (9 mg, 0.008 mmol, 0.05 equiv) for 16 h at 85 °C. Purification by FCC (CH_2_Cl_2_:EtOAc 95:5 to 80:20) and (hexanes:EtOAc 70:30) delivered benzothiazole biaryl **1d** as a pale yellow solid (40 mg, 0.11 mmol, 69%): *R*_f_ = 0.47 (CH_2_Cl_2_:EtOAc 85:15); mp 141–142 °C; ^1^H NMR (500 MHz, CDCl_3_) δ 9.02 (app s, 1H), 8.32 (app dd, *J =* 1.8, 0.6 Hz, 1H), 7.99 (app dd, *J =* 8.4, 0.6 Hz, 1H), 7.91 (app d, *J =* 2.3 Hz, 1H), 7.68 (app ddd, *J =* 8.4, 1.8, 0.5 Hz, 1H), 7.57 (app dd, *J =* 8.5, 2.3 Hz, 1H), 7.09 (app dd, *J =* 8.5, 0.3 Hz, 1H), 6.13 (ddd, *J =* 9.8, 4.1, 1.8 Hz, 1H), 5.69 (dd, *J =* 9.8, 2.1 Hz, 1H), 4.90–4.87 (m, 1H), 4.60 (dd, *J =* 6.0, 4.1 Hz, 1H), 4.44 (dd, *J =* 10.2, 5.9 Hz, 1H), 3.44 (dd, *J =* 12.4, 10.2 Hz, 1H), 3.41–3.33 (m, 1H), 2.23 (dd, *J =* 11.7, 8.0 Hz, 1H), 1.68–1.62 (m, 1H), 1.53 (d, *J =* 4.6 Hz, 1H); ^13^C{^1^H} NMR (125 MHz, CDCl_3_) δ 157.1, 154.8, 153.8, 139.3, 134.6, 133.6, 132.1, 128.2, 128.1, 127.1, 125.4, 125.0, 122.0, 121.3, 117.9, 80.8, 74.8, 73.5, 70.0, 36.7, 34.6; ESI-HRMS calculated for C_21_H_17_NO_3_SNa [M + Na]^+^ 386.0827, found 386.0820.

**Pyrimidine Biaryl 1e:** General procedure A was followed with aryl bromide **6** (50 mg, 0.16 mmol, 1.0 equiv), toluene:DME:H_2_O (0.7 mL:0.9 mL:2.0mL), pyrimidine-5-boronic acid **5e** (30 mg, 0.24 mmol, 1.5 equiv), Cs_2_CO_3_ (78 mg, 0.56 mmol, 3.5 equiv) and Pd(PPh_3_)_4_ (9 mg, 0.008 mmol, 0.05 equiv) for 16 h at 85 °C. Purification by FCC (hexanes:EtOAc 80:20 to 60:40) delivered pyrimidine biaryl **1e** as a pale yellow solid (20 mg, 0.065 mmol, 40%): *R*_f_ = 0.45 (hexanes:EtOAc 50:50); mp 183–184 °C; ^1^H NMR (500 MHz, CDCl_3_) δ 9.01 (s, 1H), 8.67 (s, 1H), 7.76 (d, *J =* 2.3 Hz, 1H), 7.31 (dd, *J =* 8.4, 2.3 Hz, 1H), 7.07 (d, *J =* 8.4 Hz, 1H), 6.12 (ddd, *J =* 9.8, 4.1, 1.8 Hz, 1H), 5.70 (dd, *J =* 9.8, 2.1 Hz, 1H), 4.83 (br s, 1H), 4.58 (dd, *J =* 6.1, 4.1 Hz, 1H), 4.48–4.43 (m, 1H), 3.44–3.35 (m, 2H), 2.23 (dd, *J =* 11.8, 8.0 Hz, 1H), 1.72 (br s, 1H), 1.67–1.59 (m, 1H); ^13^C{^1^H} NMR (125 MHz, CDCl_3_) δ 158.5, 156.8, 154.2(2), 133.8, 133.5, 128.7, 128.5, 127.7, 126.9, 126.8, 118.7, 80.7, 74.6, 73.6, 70.1, 36.7, 34.6; ESI-HRMS calculated for C_18_H_17_N_2_O_3_ [M + H]^+^ 309.1239, found 309.1220.

### 4.4. Biological Methods

**Cells and Cell Culturing Methods:***Leishmania tarentolae* (*L. tarentolae*) were cultured at 22 °C in the dark in 250 mL CELLTREAT Suspension Culture Flasks (ThermoFisher Scientific, Waltham, MA, USA). Brain heart infusion media (BHI) supplemented with penicillin, streptomycin, and hemin was used for *L. tarentolae* culturing following the method of Morgenthaler et al. [97]. Axenic *Rattus norvegicus* C6 glioma cells (ATCC CCL-107) were cultured in FALCON 6-well culture plates using high glucose Dulbecco’s Modified Eagles Medium (incomplete DMEM; Sigma Life Sciences D6429; St. Louis, MO, USA) supplemented with 15% (*v*/*v*) horse serum (ATCC; Manassas, VA, USA) and 5% (*v*/*v*) heat-treated fetal bovine serum (GIBCO; Waltham, MA, USA) (now designated as complete DMEM) at 37 °C and maintained under a 5% CO_2_ atmosphere following the method of Vallejo et al. [98]. For experimentation, cells were harvested by trypsinization, following the manufacturer’s recommendations (Sigma Aldrich), and resuspended in complete DMEM. This cell suspension was then added to sterile 96-well cell culture plates (Falcon^®^) in 100 μL aliquots. MTT viability assay [95] was performed following the method of Mendez et al. [99]. From a homogenous suspension, *Leishmania* (100 µL per well) were loaded in quadruplicate; for glial cells, the growth medium was removed, replaced with 100 µL per well of incomplete medium diluted 1:10 with saline. Reactions were evaluated at A 595 nm using a BIO-RAD iMark microplate reader. Incubation times were varied depending on stage in the growth curve and number of cells, as the MTT assay is dependent upon NAD(P)H-dependent oxidoreductase enzymes [100]. MTT samples were averaged per condition, corrected for BHI background, and normalized per hour of MTT incubation at room temperature (following the method of Mendez et al. [99]). When applicable, ANOVA followed by Tukey Post Test was used to evaluate statistical differences relative to control cells; a *p* value < 0.05 is considered significantly different. Number of replicates are indicated in the appropriate graphs and tables.

**Evaluation of Compound Effects on *L. tarentolae* Growth:** Equal volumes of early log-phase *L. tarentolae* were aliquoted into 4.95 mL of BHI medium in separate 25 mL Falcon culture flasks. The 30 mM stock solutions were diluted 1:10 in DMSO for a working stock of 3 Mm. Then, 50 µL of the working stock was added to each flask for a final concentration of 30 µM of compound and 1% DMSO. A no-cell flask, containing BHI and 1% DMSO, was used as the blank and a cell culture containing 1% DMSO was used as the control cells. The MTT assay was performed before compound addition to test baseline cell viability and then every 24 h following additions for 96 h.

**Evaluation of *Leishmania* Secreted Acid Phosphatase (SAP):***Leishmania* secreted acid phosphatase (SAP) assay (using the method of Mendez et al. [99]) was used to determine enzyme activity from cell-free supernatant as a function of compound incubation. Homogenous samples were taken from culture flasks of varying conditions and stock cultures and centrifuged (10,000 rpm, 60 s, Eppendorf Centrifuge 5415C) in 1.5 mL polypropylene tubes, and then, the supernatant was used as the enzyme source. Per each reaction, supernatant (450 µL) was mixed with 450 µL of sodium acetate buffer (0.5 M, pH 4.5) in a 1.5 mL microcentrifuge tubes and reactions were done in triplicate (following the method of Mendez et al. [44]). One hundred µL of a 5 mg/mL (14.7 mM) solution of para-nitrophenyl phosphate disodium salt hexahydrate (pNPP, Sigma-Aldrich) was added to each tube to start the enzymic reaction. The reaction was stopped ~24 h after it started with the addition of 100 µL of NaOH (10.0 M). SAP enzymic activity produces *para*-nitrophenol, which is converted to the nitrophenylate anion upon the addition of NaOH, it has an absorbance at 405 nm, and it was evaluated using an Agilent Hewlett Packard 8453 UV-visible spectrophotometer. Samples were averaged per condition for *n* = 4 replicates, corrected for BHI, and normalized per 24-h incubation time.

**Evaluation of Direct Enzymic Effect of Test Compounds on SAP:** A stock culture of *L. tarentolae* in the late stationary phase was centrifuged (3000 rpm, 15 min @ 7 °C, Labnet HERMLE Z 400K centrifuge), and the cell-free supernatant was collected. This standard pool of enzyme was separated into equal volumes and allowed to incubate with each of the 6 compounds at 30 µM or 1% DMSO for 15 min, 18 h, or 46 h before starting the SAP assay.

**SAP Enzyme Kinetic Assay using Selected Test Compounds (following the method of Dorsey et al**. [28]): A stock culture of *L. tarentolae* in the late stationary phase, was pelleted, retaining the supernatant. This standard pool of SAP enzyme was separated into equal volumes and allowed to incubate with 30 µM compounds **6**, **1c**, and **1e**. Pre-incubated samples were aliquoted with 13 varying substrate concentrations ranging from 4.5 µM to 1787 µM.

**Evaluation of Test Compound Acute Effects on Glial Viability:** Test compounds prepared in DMSO and diluted into incomplete DMEM were introduced, at a concentration of 30 µM, when the glial cells in the 96-well plates reached confluency. MTT assays were conducted in incomplete DMEM diluted 1:10 with sterile saline following a two-hour incubation with the compounds.

**Effective Dose (ED-50) Study on *L. tarentolae:*** Equal volumes of late log-phase *L. tarentolae* were aliquoted into separate flasks with compounds **6**, **1c**, or **1e** at 8 varying final concentrations ranging from 3 to 200 µM. MTT assays were performed 24 h and 48 h post-addition of compounds.

**Evaluation of Photosensitivity of *L. tarentolae* in the Presence of Test Compounds:** Equal volumes of late log-phase *L. tarentolae* were aliquoted into separate flasks with 30 µM of compounds **6**, **1c**, or **1e**. Cultures were incubated 1 h in the dark to allow uptake of compounds before exposing to a fluorescent bulb (following the method of Morgenthaler et al. [97]), or a UV bulb for 1 h. Cells kept in the dark were considered the control cells. MTT assays were performed directly after compound addition as well as 2 h, 24 h, and 48 h after light exposure.

**Evaluation of production of nitric oxide by *Leishmania* in the presence of test compounds:** Nitric oxide (NO) assay was performed using DAF-FM diacetate (a compound that fluoresces after reaction with nitric oxide; from Millipore Sigma, Darmstadt, Germany) at a final concentration of 20 μM and 1% DMSO in saline. Cells were centrifuged (10,000 rpm, 60 s, Eppendorf Centrifuge 5415C) and resuspended in this DAF-FM diacetate solution for 1 h before re-centrifuging, which was followed by washing the cell pellet with 500 µL saline. Cells were resuspended in a final total volume of saline such that there were a similar number of cells per sample. Prior to imaging, 10 μL of each sample was loaded onto a glass slide within a silicon grease ring and then covered with a cover slip. Sample slides were placed in a BZX-810 Keyence Fluorescent Microscope for imaging using a green fluorescent protein filter (GFP; 470 nm: 525 nm excitation-emission coupling). Images were captured at a 1.5 s exposure time and overlays of fluorescent cells with the brightfield images were produced using the analyzer software. Cells were then counted for number of green fluorescent cells (indicating production of nitric oxide) and number of non-green cells.

## Figures and Tables

**Table 1:**
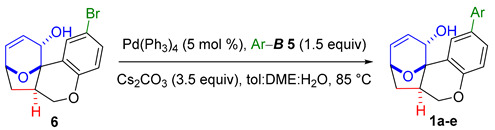
Suzuki-Miyaura cross-coupling with aryl bromide **6.**

Entry	Ar-*B* (5)	% Yield (1) *^a^*
1	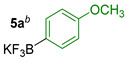	69 (**1a**)
2	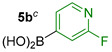	38 (**1b**)
3	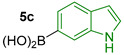	19 (**1c**)
4	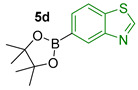	69 (**1d**)
5	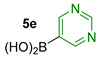	40 (**1e**)

*^a^* Isolated yield. *^b^* 1.2 equiv. of Ar-***B* 5a** was utilized. *^c^* Alternative conditions were utilized: Pd(dppf)Cl_2_ (30 mol %), aq. K_2_CO_3_ (6.0 equiv) in tol:EtOH (3:1) at 110 °C.

**Table 2:** Slopes of growth phase curve (0–72 h) and recovery phase curve (96–144 h).

	Compound(s) Added								
Condition	1% DMSO	**6**	**1a**	**1b**	**1c**	**1d**	**1e**	**1b/1e**	**1c/1e**
Growth phase (AU/hr)	0.013	0.013	0.0071	0.012	0.0042	0.0089	0.013	0.012	0.0023
Recovery phase (AU/hr)	0.017	0.018	0.012	0.016	0.0059	0.014	0.016	0.016	0.0042
Recovery/Growth Phase Ratio	1.30	1.37	1.73	1.30	1.43	1.55	1.26	1.37	1.85

**Table 3:** Calculated SAP K_M_ and V_max_ (from Lineweaver-Burk Plots) after 24-h incubation.

	6	1c	1e	DMSO Control
K_M_ (µM)	386	421	364	396
V_max_ (A 405 nm /24 hr)	1.07	1.15	1.06	1.08

**Table 4:** Cell counts for microscopic images of DAF-FM diacetate incubated *L. tarentolae* and calculated percent of cells able to express nitric oxide (green).

	Compound(s) Added								
Cell counts	1% DMSO	**6**	**1a**	**1b**	**1c**	**1d**	**1e**	**1b +1e**	**1c +1e**
cells green	488	344	423	227	525	438	56	69	282
cells not green	248	494	674	669	850	703	315	323	334
Total cells counted	736	838	1097	896	1375	1141	371	392	616
% total green	66.3	41.1	38.6	25.3	38.2	38.4	15.1	17.64	45.8

**Table 5:** Glial cell MTT average absorbances relative to 1% DMSO cell control (*n* = 4).

	6	1a	1b	1c	1d	1e	DMSO Control
% DMSO Control	95.4	129.6	114.8	116.9	94.4	97.1	100

**Table 6:** Calculated ED-50 concentration for **1c** and **1e** at 24 and 48 h after addition.

Time after Addition	6 ED-50 (µM)	1c ED-50 (µM)	1e ED-50 (µM)
24 h	>200	72.4	23.1
48 h	>200	24.9	31.5

**Table 7:** MTT absorbances relative to 1% DMSO cell control, **6**, **1c**, or **1e** at 2, 24, and 48 h post addition. D = DMSO, N = normal conditions (dark), F = fluorescent bulb, UV = ultraviolet bulb.

Time after Addition	D-N	D-F	D-UV	6-N	6-F	6-UV	1c-N	1c-F	1c-UV	1e-N	1e-F	1e-UV
2 h	100	99.7	99.8	99.2	101.5	102.7	104.9	102.7	98.2	98.2	95.0	95.8
24 h	100	91.1	96.6	98.0	103.3	105.1	73.8	75.0	70.9	98.0	97.1	94.9
48 h	100	104.0	100.6	99.6	105.5	100.3	64.0	67.6	61.6	99.3	100.0	100.1

## Supplementary Materials

The following are available online, Figure S1: 1H NMR (500 MHz) of **7** in CDCl3, Figure S2: ^13^C NMR (100 MHz) of **7** in CDCl_3_, Figure S3: ^1^H NMR (500 MHz) of **6** in CDCl_3_, Figure S4: ^13^C NMR (125 MHz) of **6** in CDCl_3_, Figure S5: ^1^H NMR (400 MHz) of **1a** in CDCl_3_, Figure S6: ^13^C NMR (100 MHz) of **1a** in CDCl_3_, Figure S7: ^1^H NMR (500 MHz) of **1b** in CDCl_3_, Figure S8: ^13^C NMR (125 MHz) of **1b** in CDCl_3_, Figure S9: ^1^H NMR (500 MHz) of **1c** in CDCl_3_, Figure S10: ^13^C NMR (125 MHz) of **1c** in CDCl_3_, Figure S11: ^1^H NMR (500 MHz) of **1d** in CDCl_3_, Figure S12: ^13^C NMR (125 MHz) of **1d** in CDCl_3_, Figure S13: ^1^H NMR (500 MHz) of 1e in CDCl_3_, Figure S14: ^13^C NMR (125 MHz) of **1e** in CDCl_3_, Figure S15. Overlay image of 1% DMSO control cell pool, Figure S16. Overlay image of compound **1b** incubation, Figure S17. Overlay image of compound **1c** incubation, Figure S18. Overlay image of compound **1e** incubation, Figure S19. Overlay image of mixed incubation of compounds **1b** + **1e**, Figure S20. Overlay of mixed incubation of compounds **1c** + **1e**.
