# Supplementary material for: Synthesis of Polycyclic Ether-Benzopyrans and In Vitro Inhibitory Activity against Leishmania tarentolae
Source: Molecules. 2020 Nov 21;25(22):5461. doi: 10.3390/molecules25225461 (PMC7700287; doi:10.3390/molecules25225461)
Supplement: Supplementary file 1 [file molecules-25-05461-s001.pdf]

Synthesis of polycyclic ether-benzopyrans and *in vitro* inhibitory activity against *Leishmania tarentolae*

Sarita Singh, Jacob P. Grabowski, Shilpa Pohani, C. Fiore Apuzzo, David C. Platt, Marjorie A. Jones and T. Andrew Mitchell**

Department of Chemistry, Illinois State University, Campus Box 4160, Normal, IL 61790-4160;

mitchell@ilstu.edu

Supporting Information

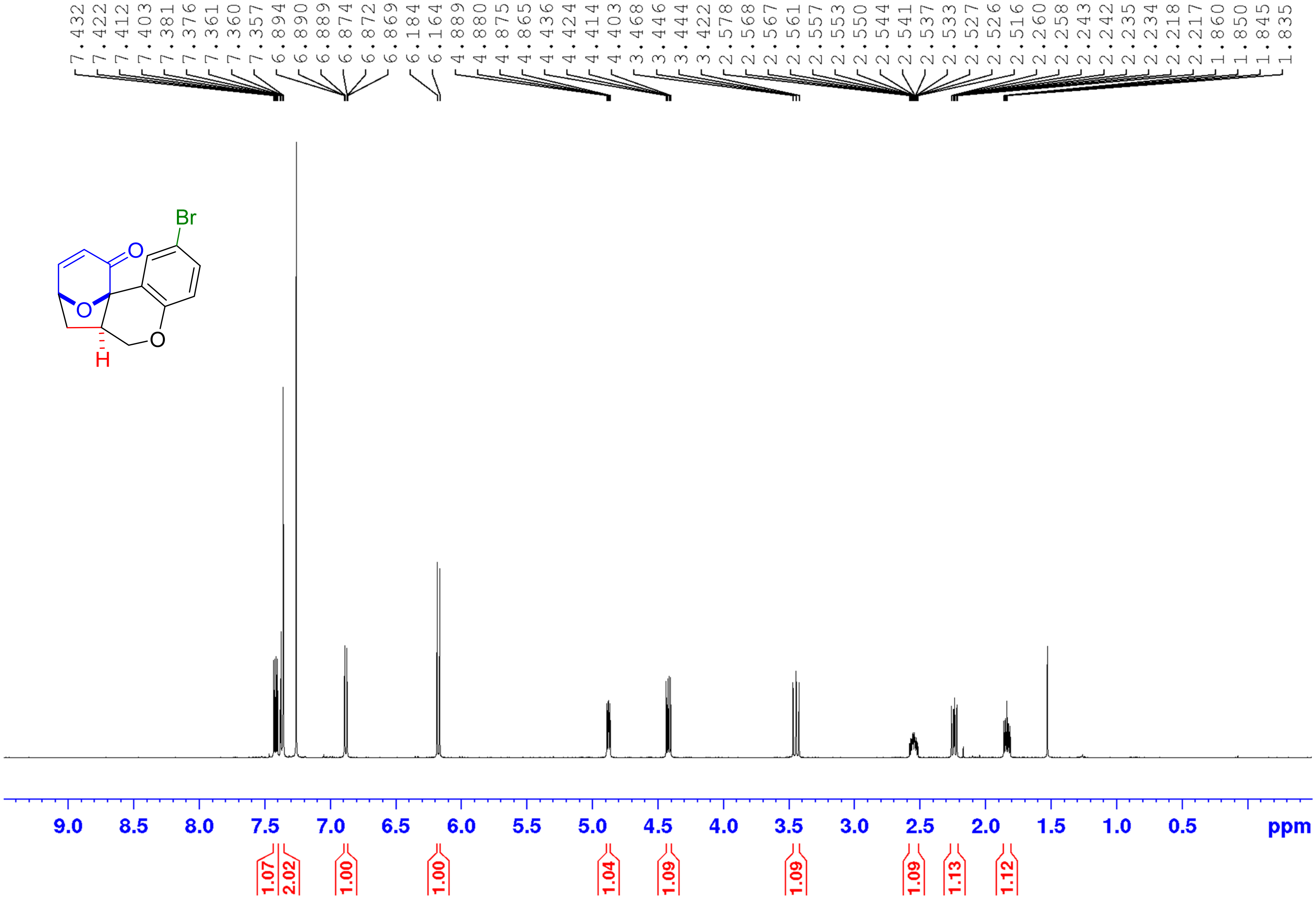

Figure S1: ^{1}H NMR (500 MHz) of 7 in $CDCl_3$

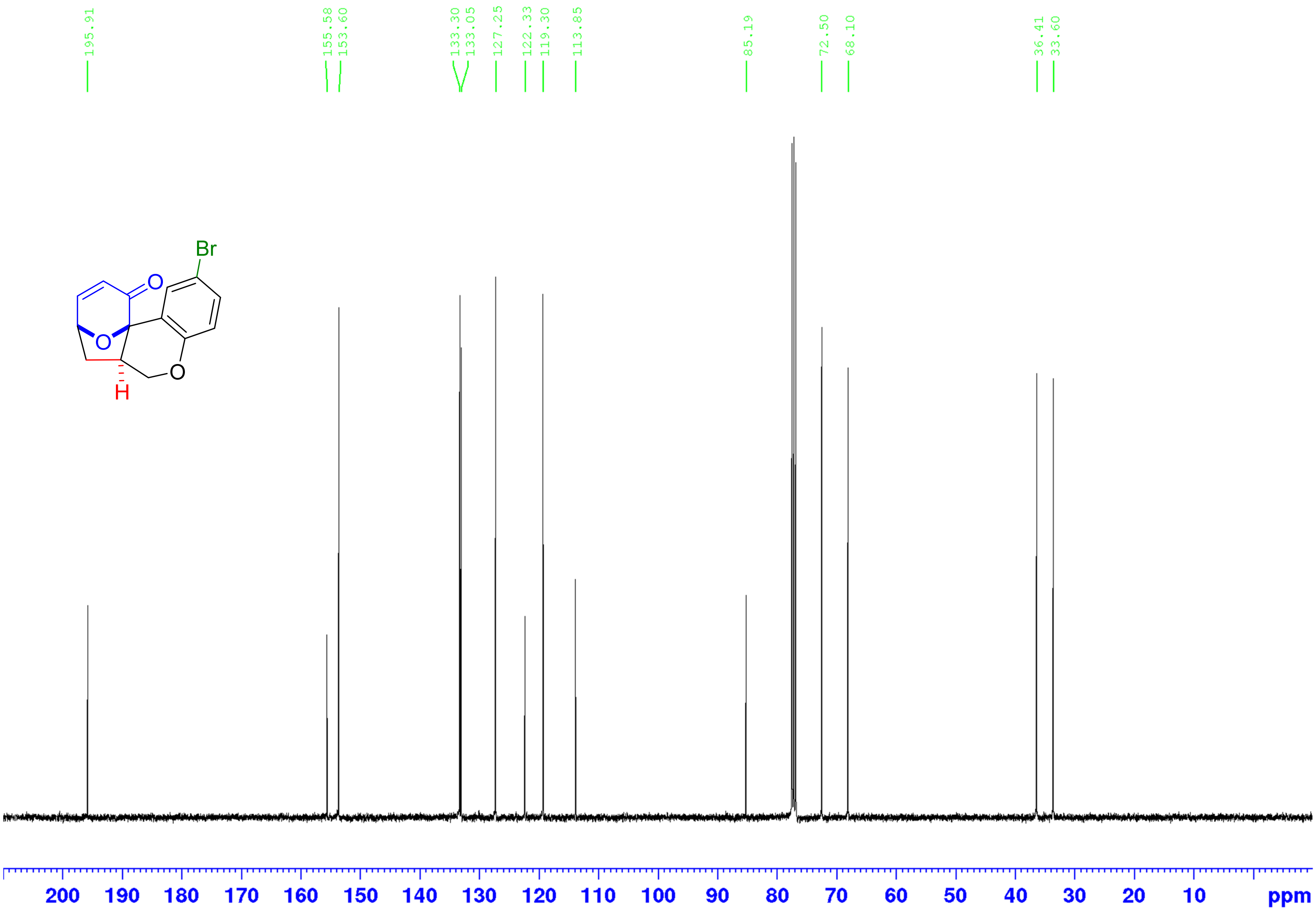

Figure S2: ^{13}C NMR (100 MHz) of 7 in $CDCl_3$

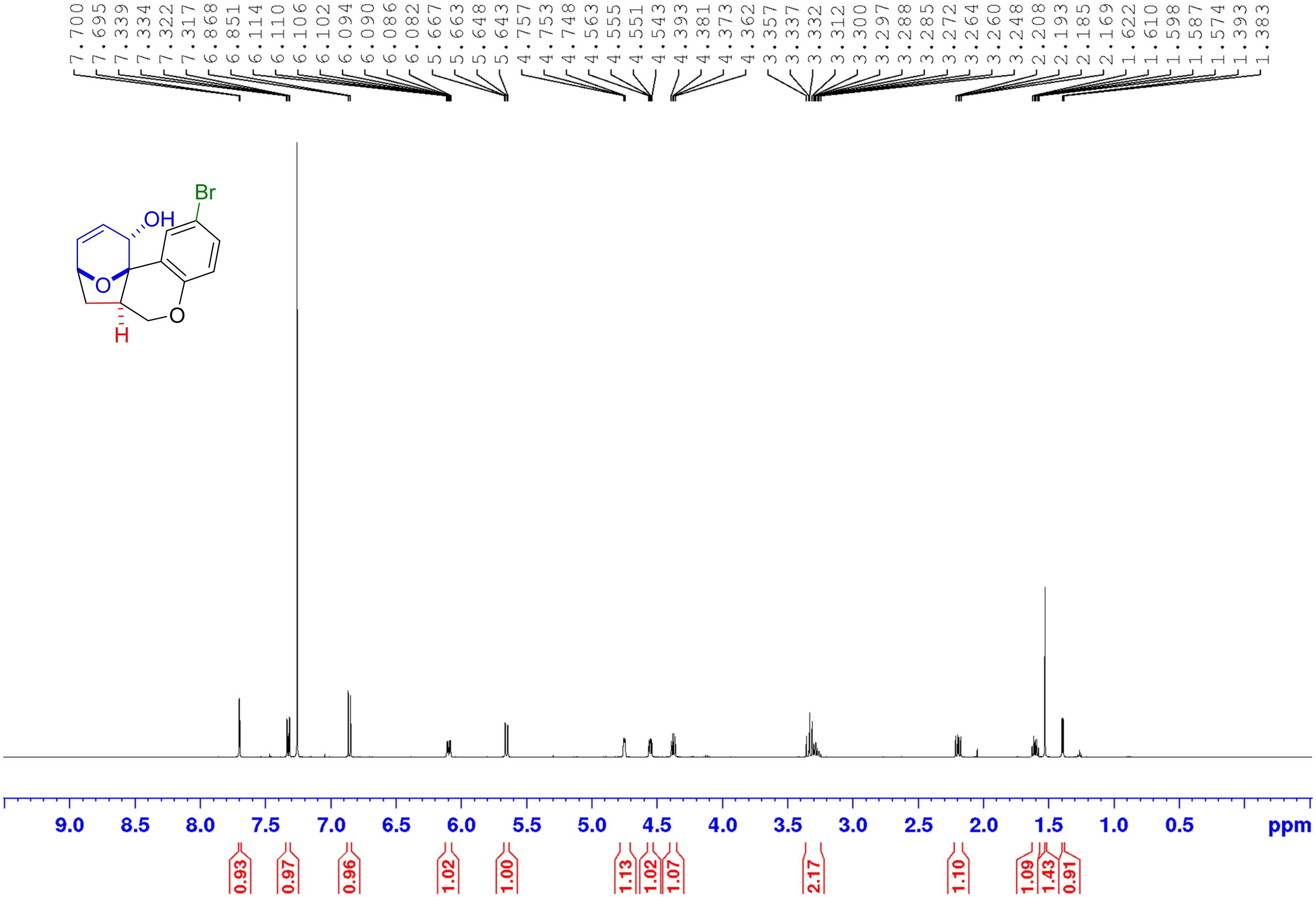

Figure S3: ^{1}H NMR (500 MHz) of 6 in $CDCl_3$

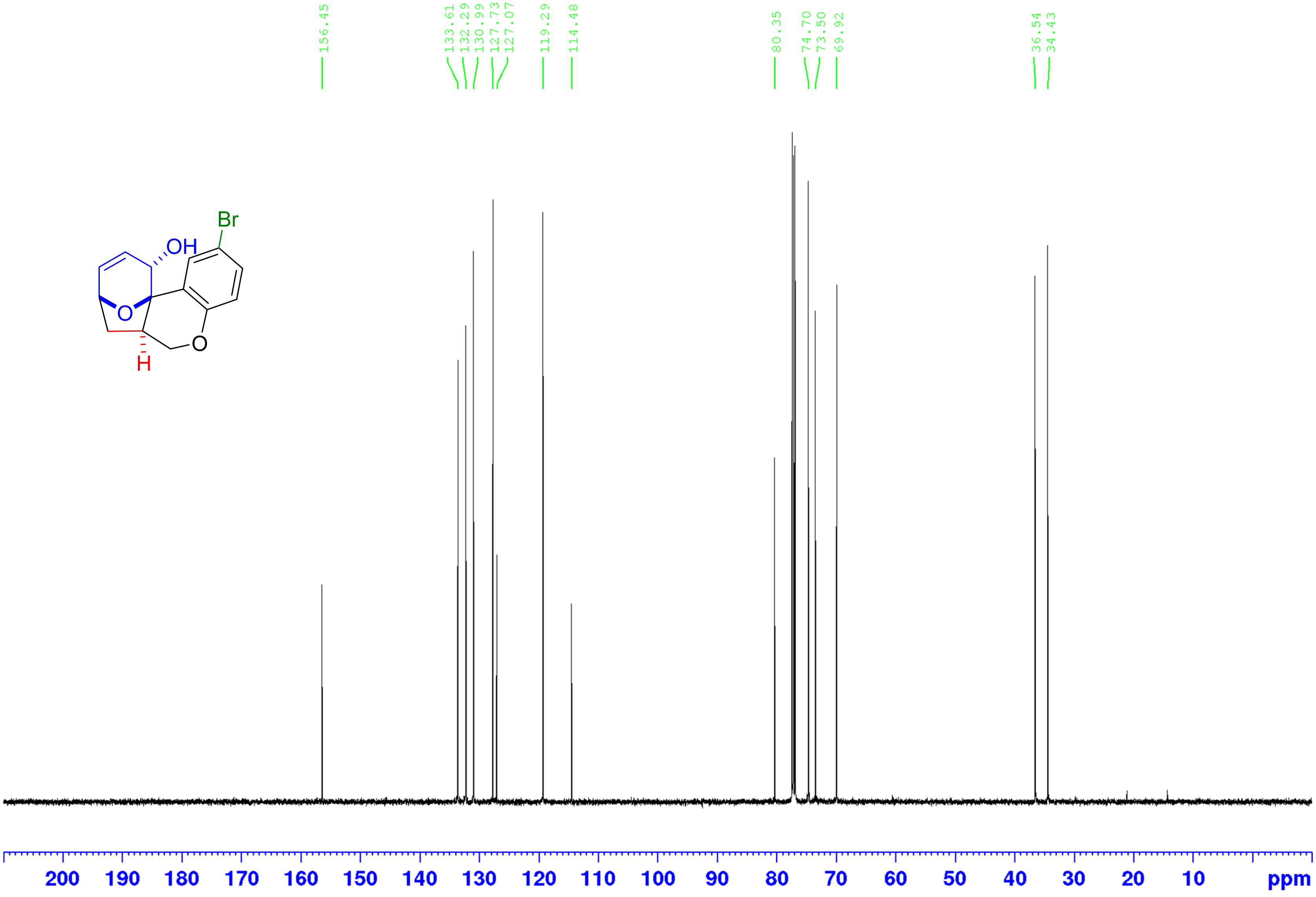

Figure S4: ^{13}C NMR (125 MHz) of 6 in $CDCl_3$

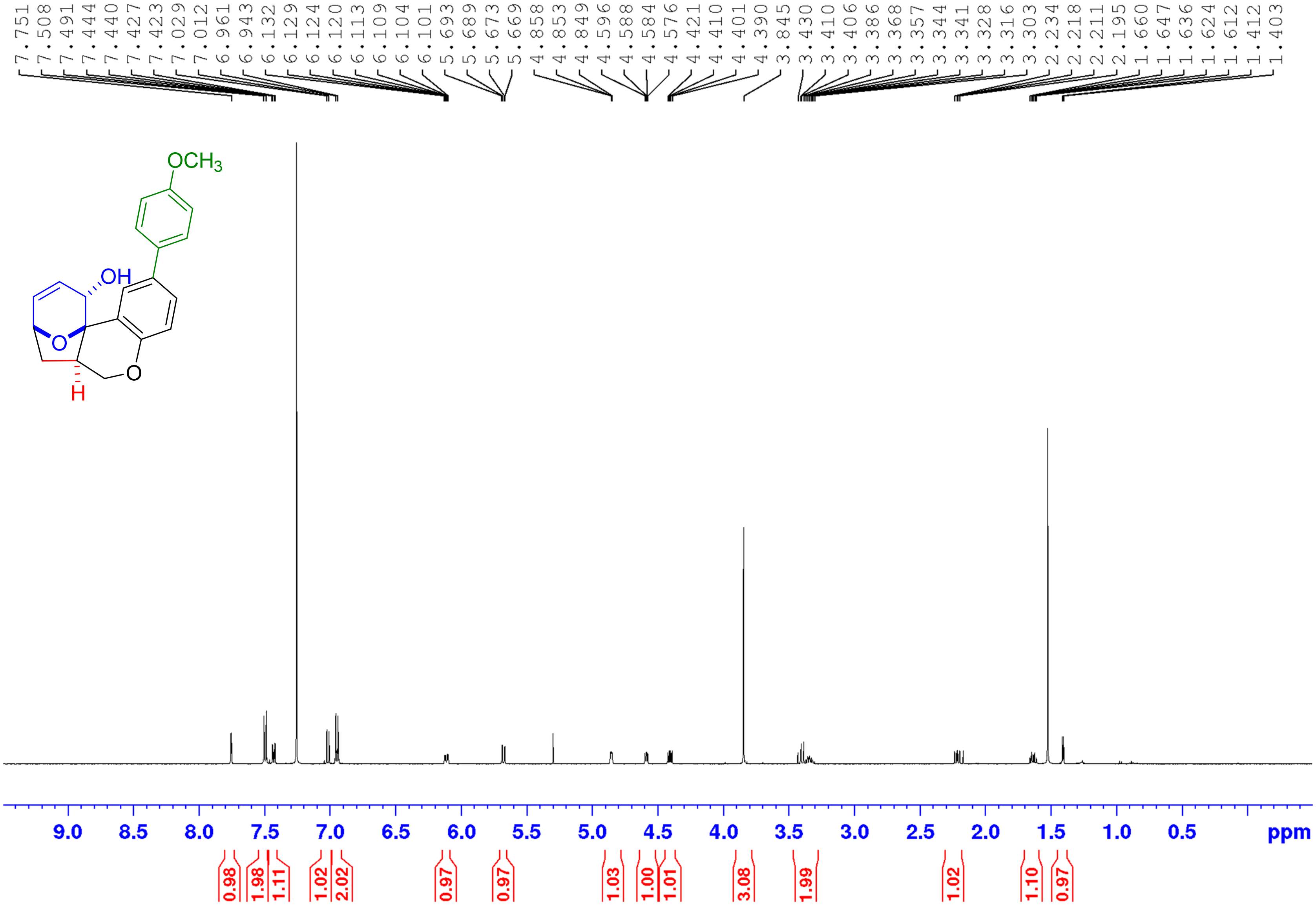

Figure S5: ^{1}H NMR (400 MHz) of 1a in $CDCl_3$

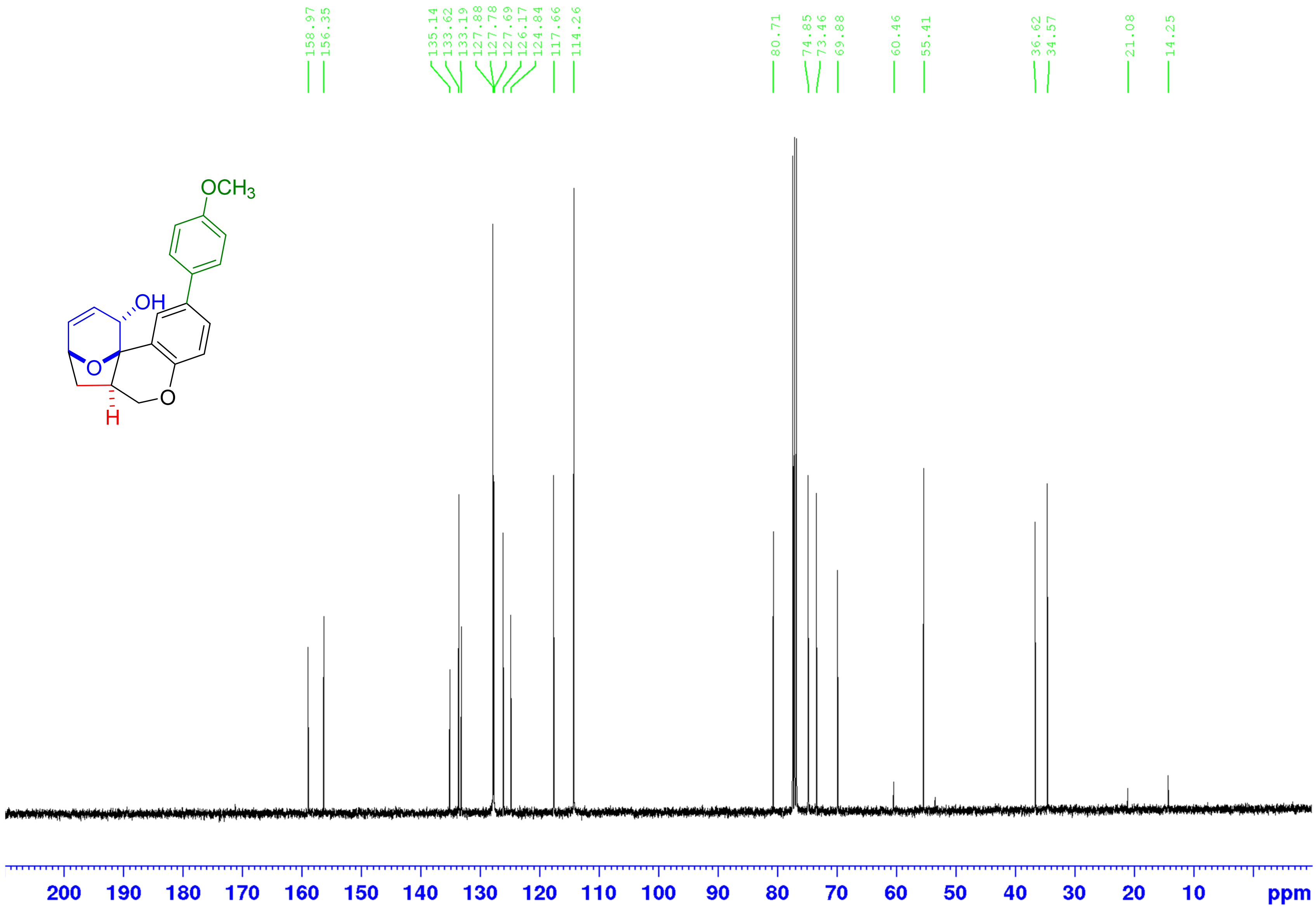

Figure S6: ^{13}C NMR (100 MHz) of 1a in $CDCl_3$

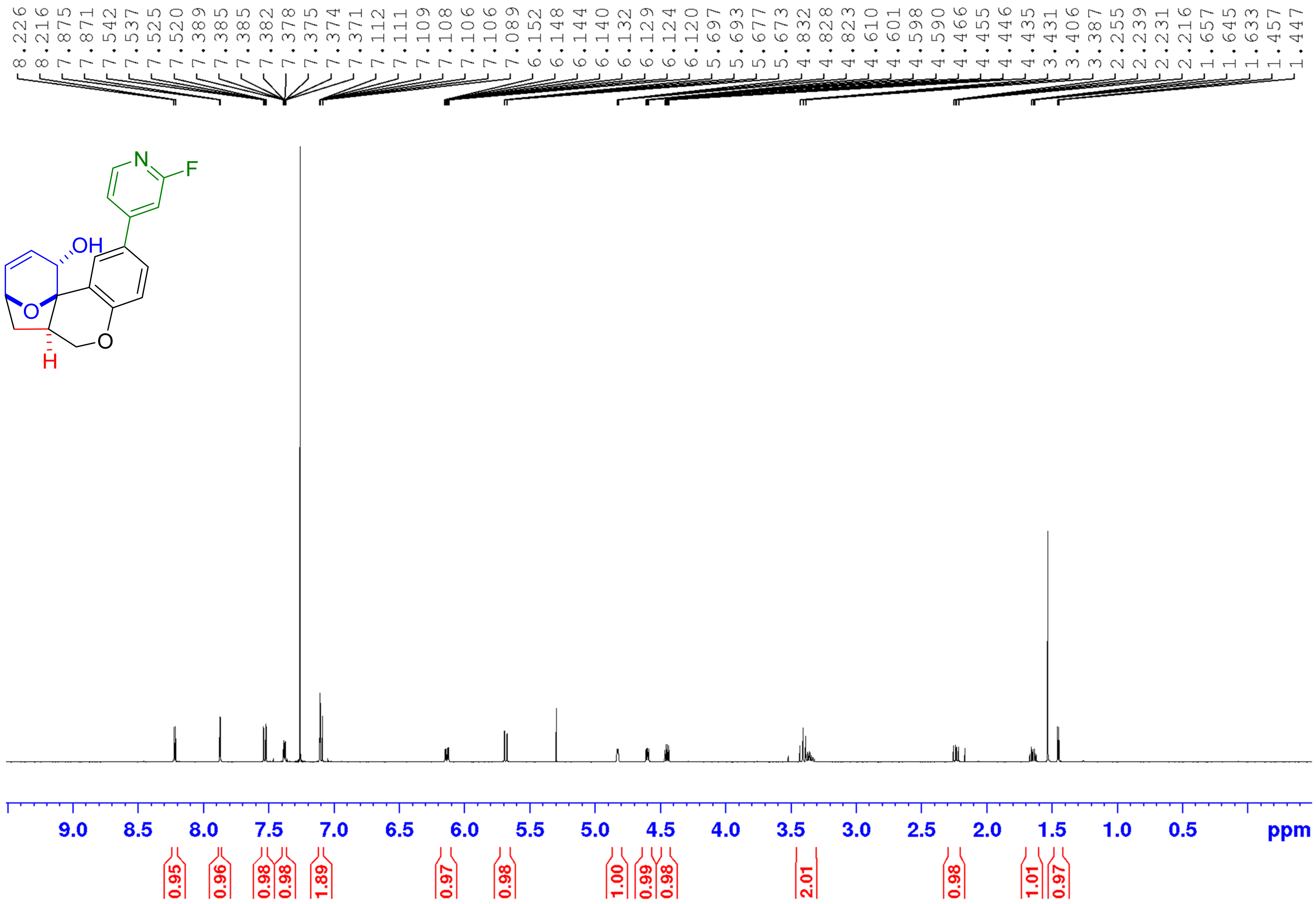

Figure S7: ^{1}H NMR (500 MHz) of 1b in $CDCl_3$

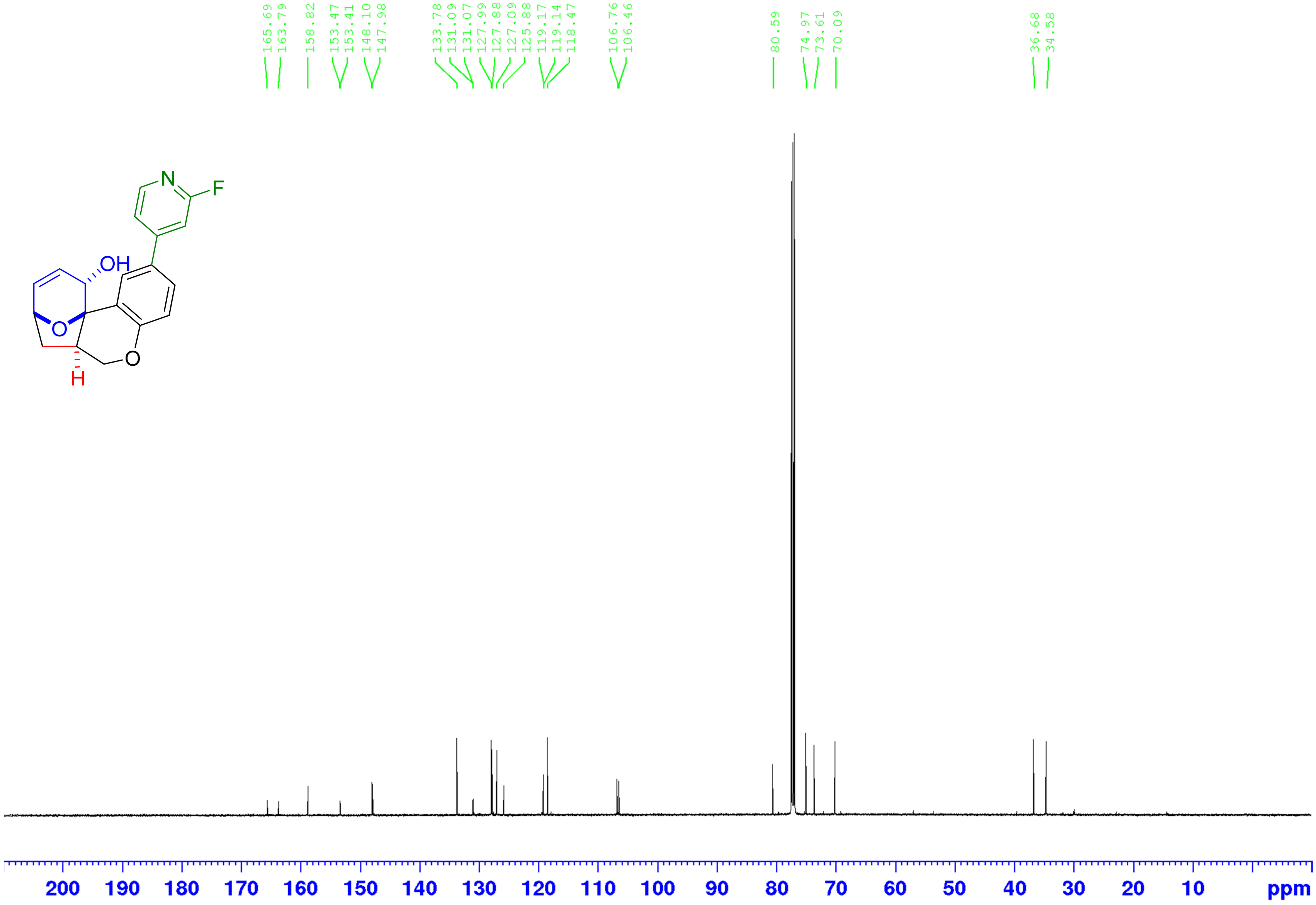

Figure S8: ^{13}C NMR (125 MHz) of 1b in $CDCl_3$

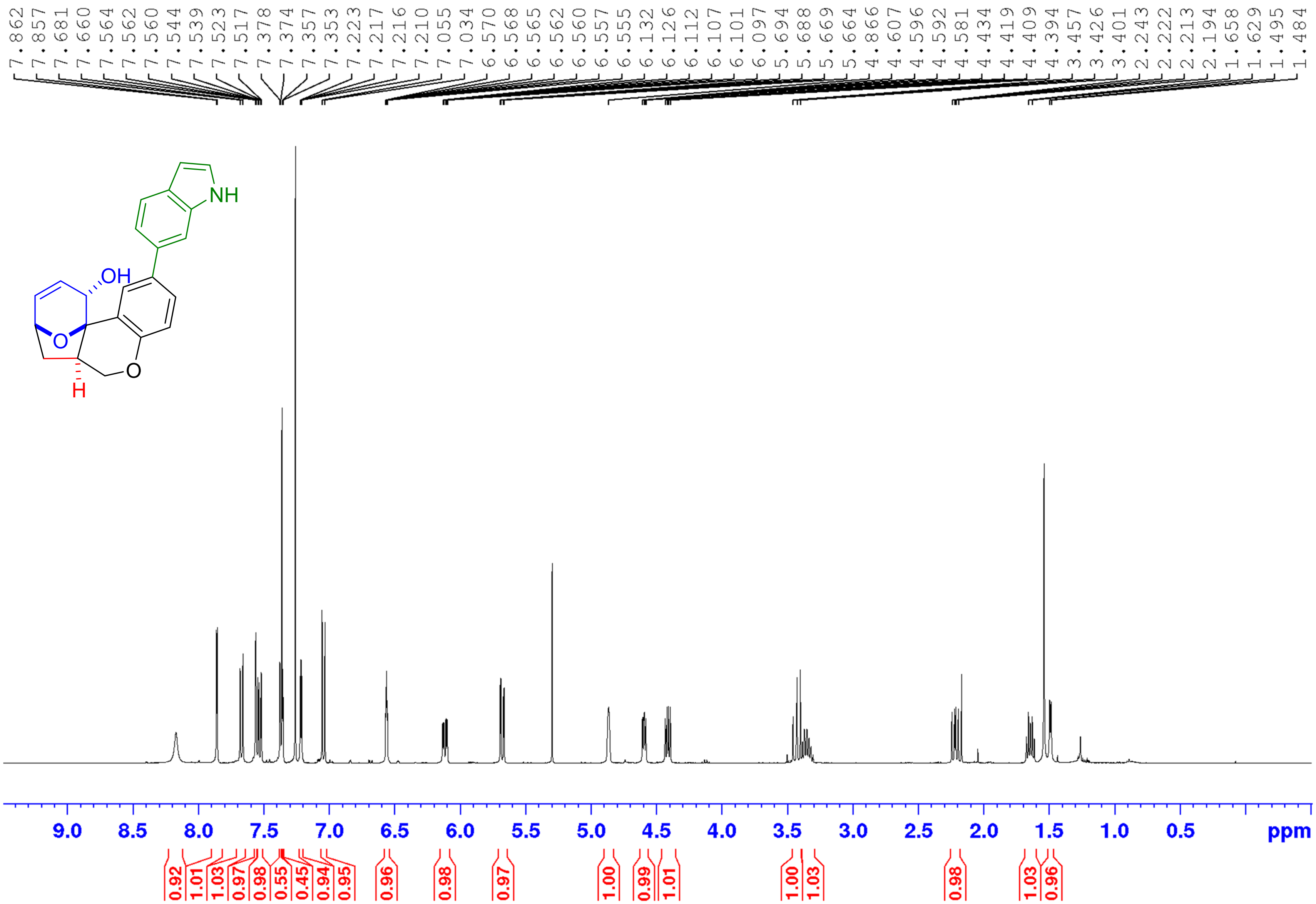

Figure S9: ¹H NMR (500 MHz) of 1c in $CDCl_3$

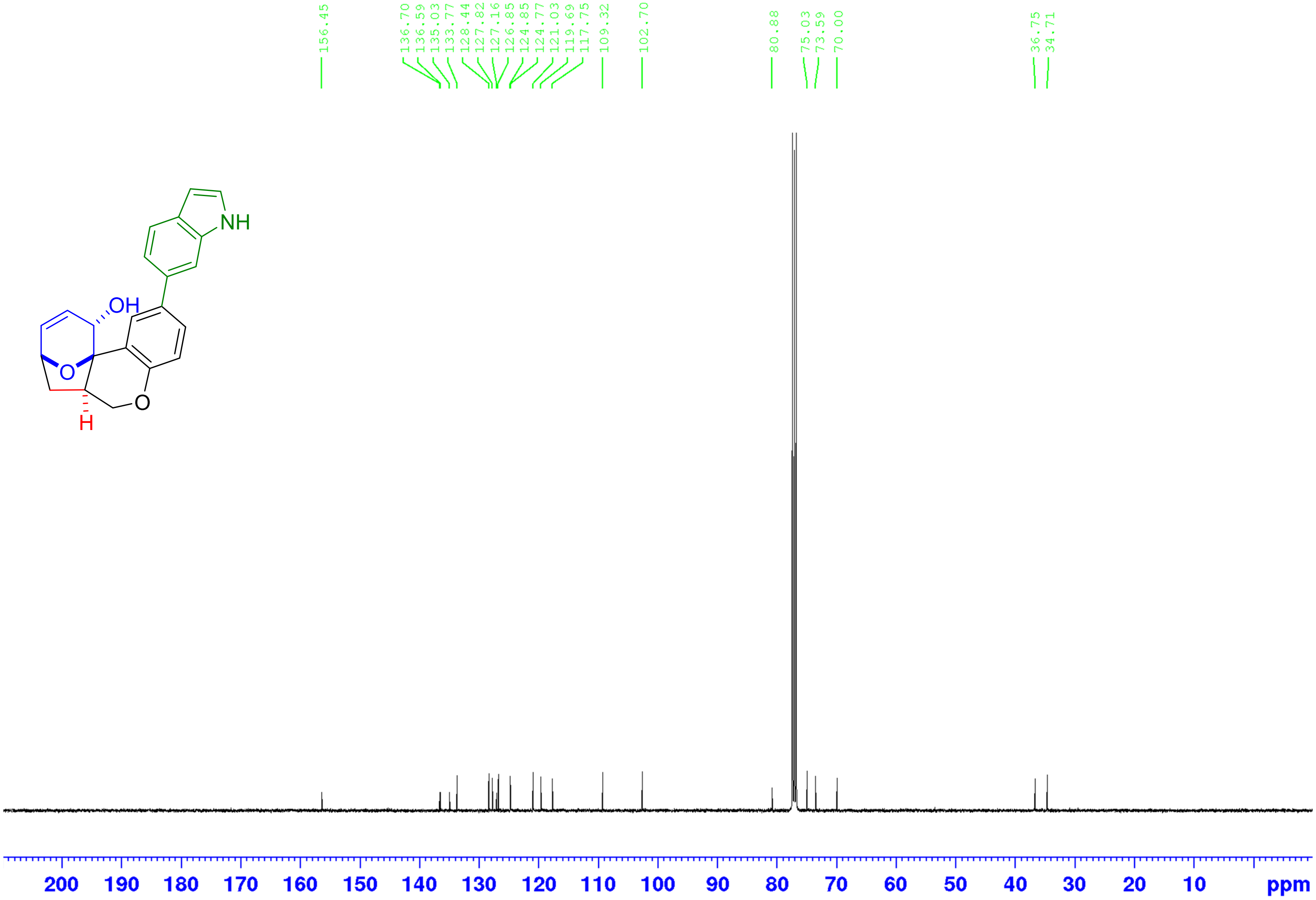

Figure S10: ^{13}C NMR (125 MHz) of 1c in $CDCl_3$

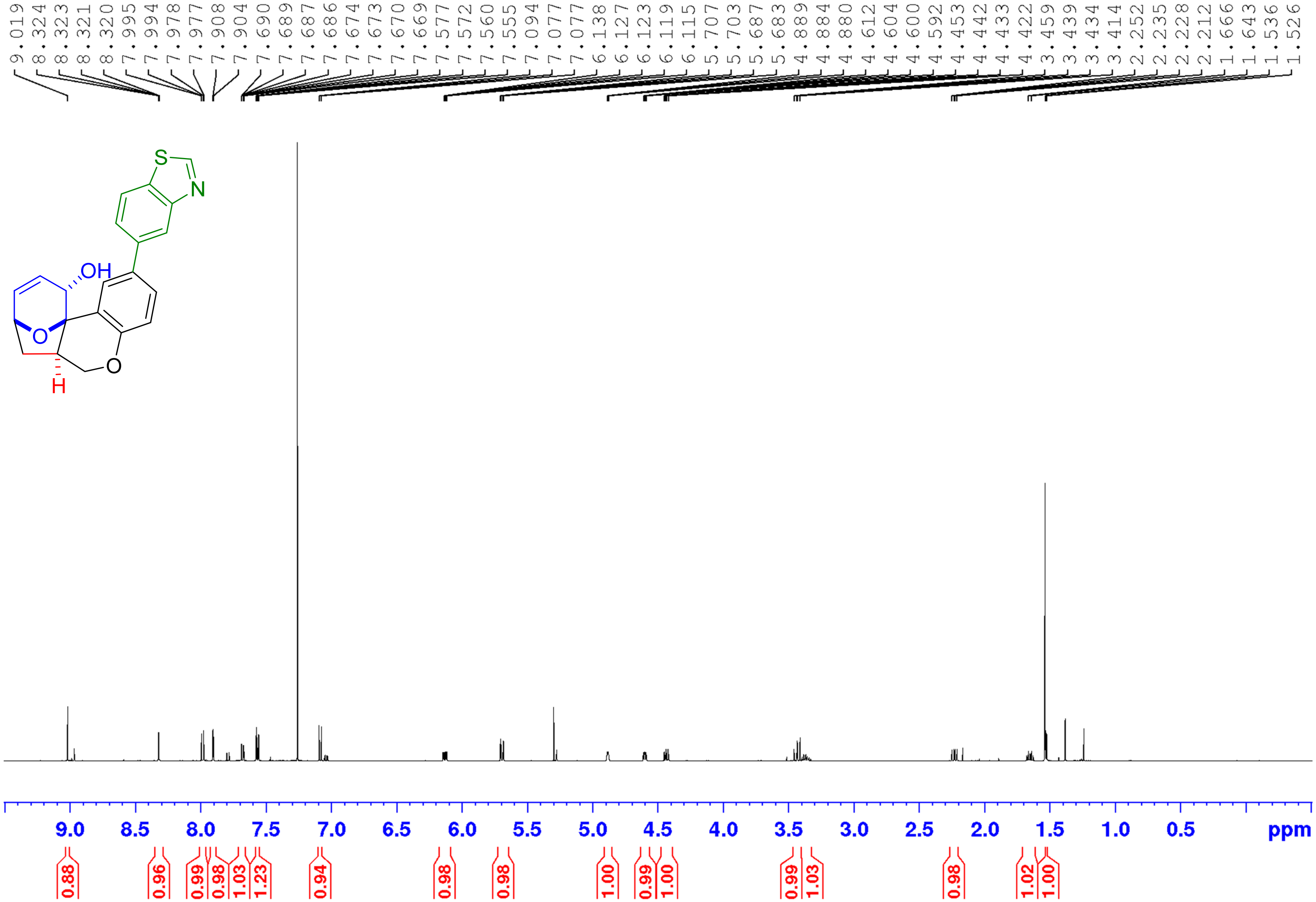

Figure S11: ^{1}H NMR (500 MHz) of 1d in $CDCl_3$

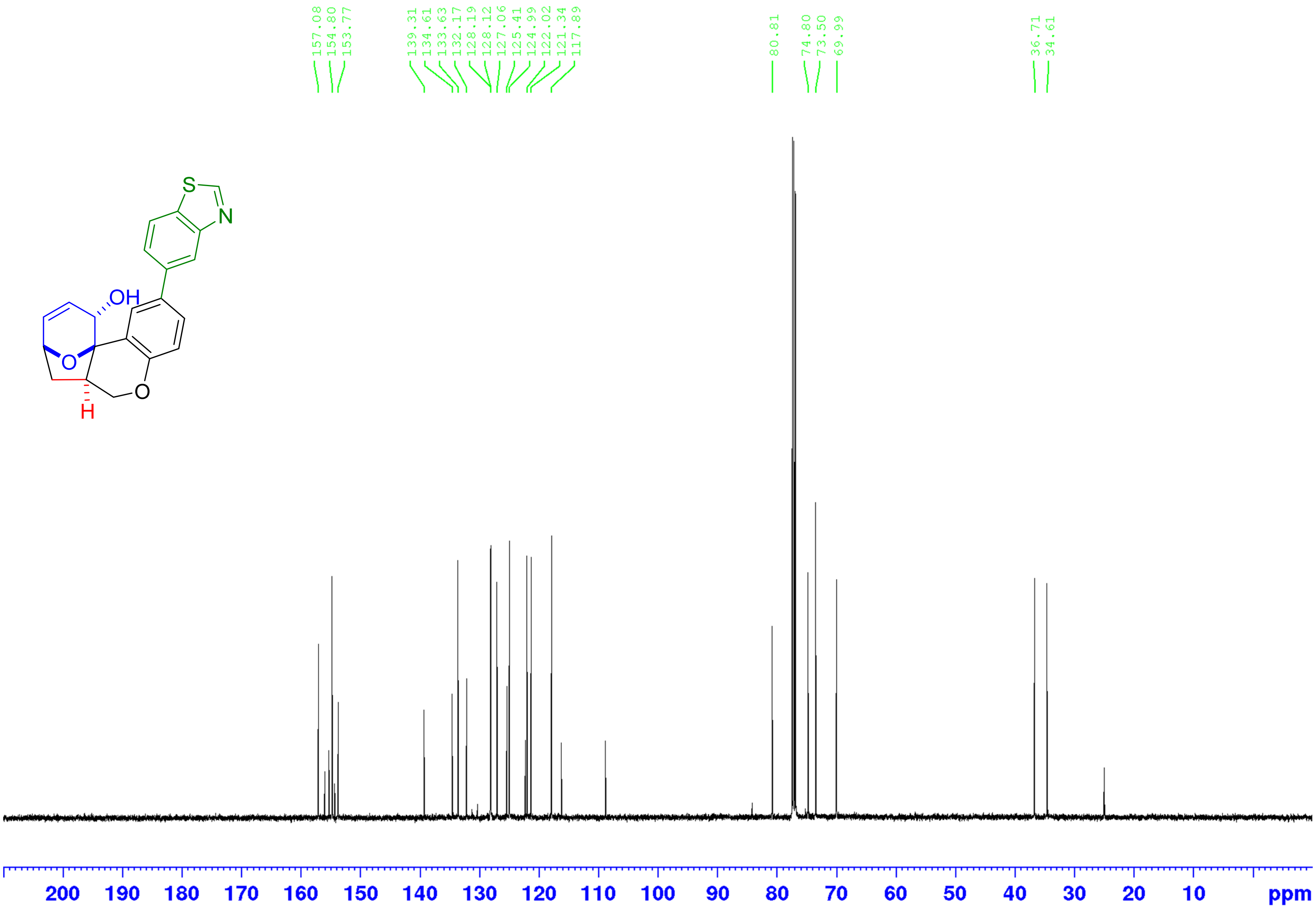

Figure S12: ^{13}C NMR (125 MHz) of 1d in $CDCl_3$

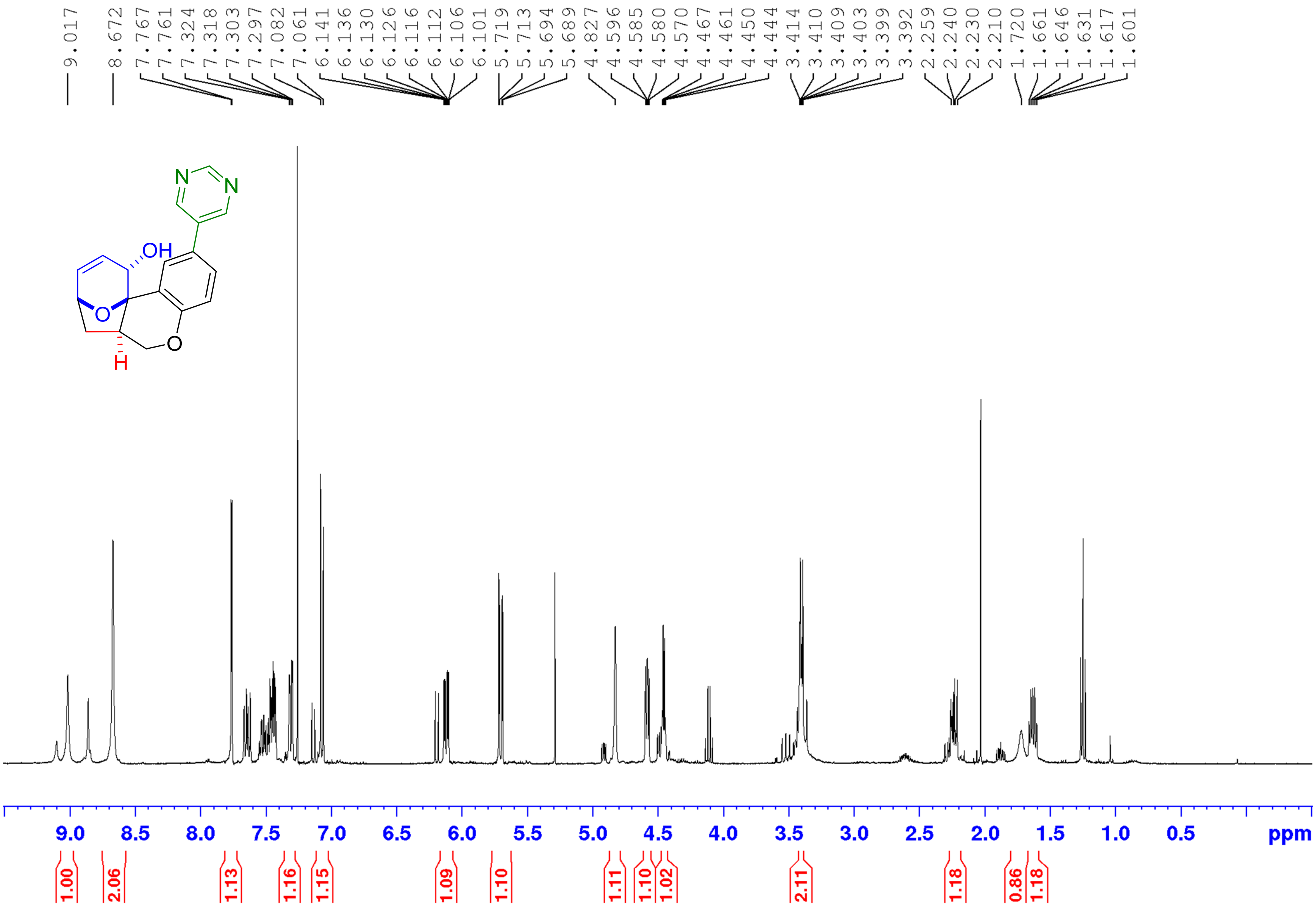

Figure S13: ^{1}H NMR (500 MHz) of 1e in $CDCl_3$

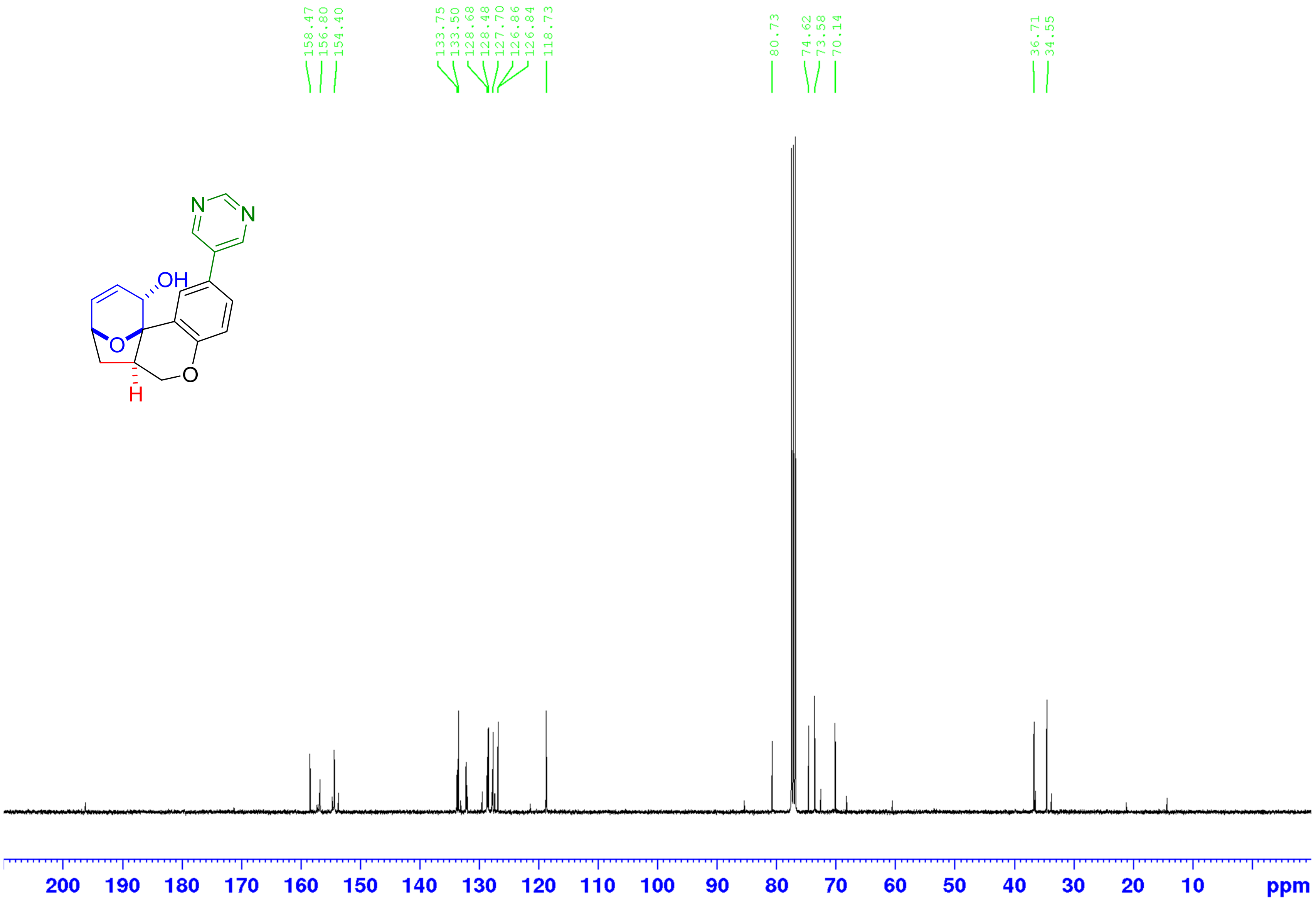

Figure S14: ^{13}C NMR (125 MHz) of 1e in $CDCl_3$

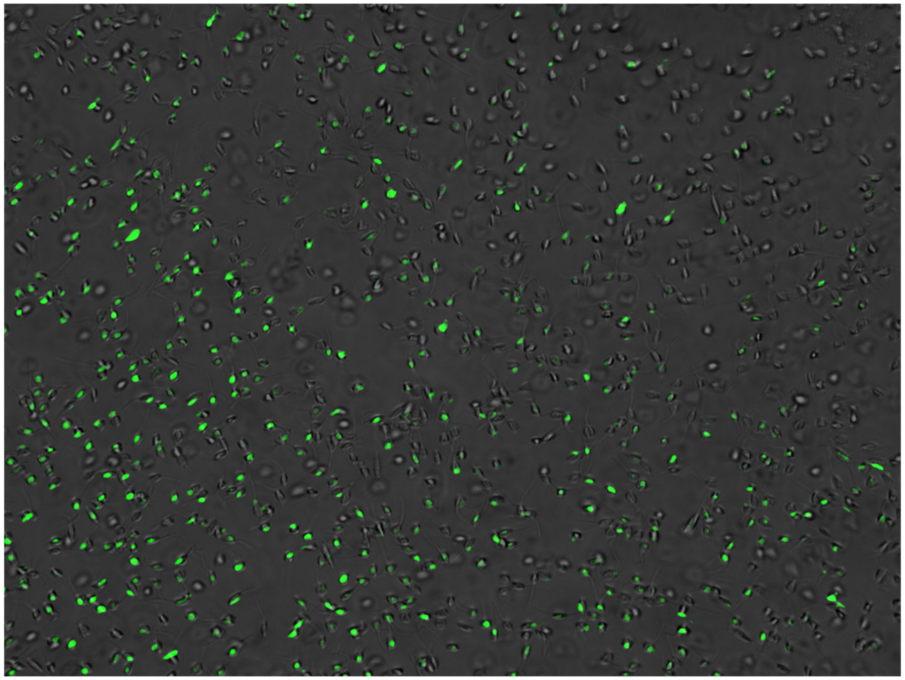

Figure S15. Overlay image of 1% DMSO control cell pool.

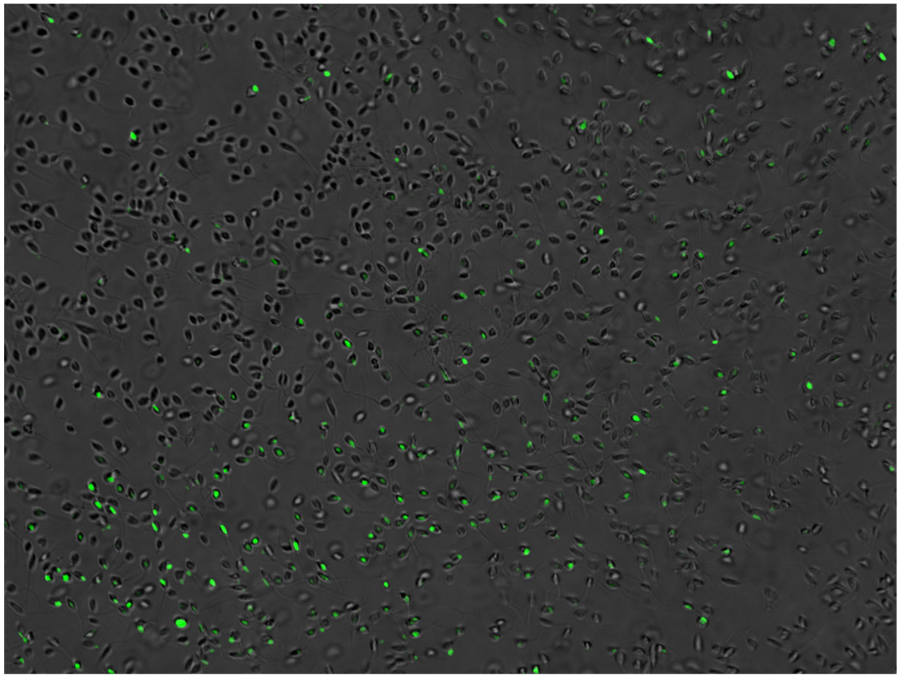

Figure S16. Overlay image of compound **1b** incubation.

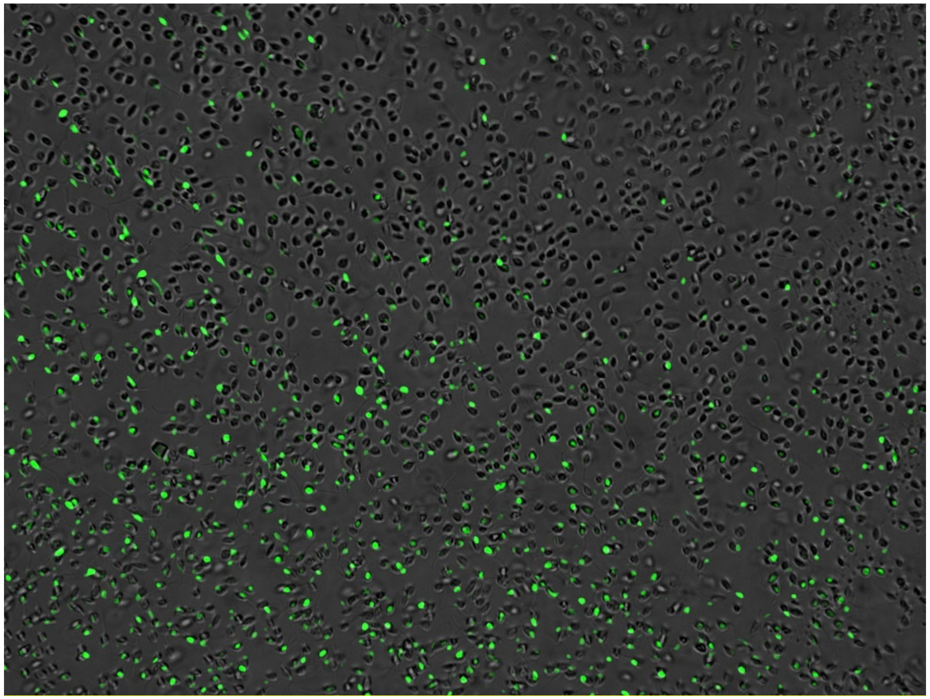

Figure S17. Overlay image of compound **1c** incubation.

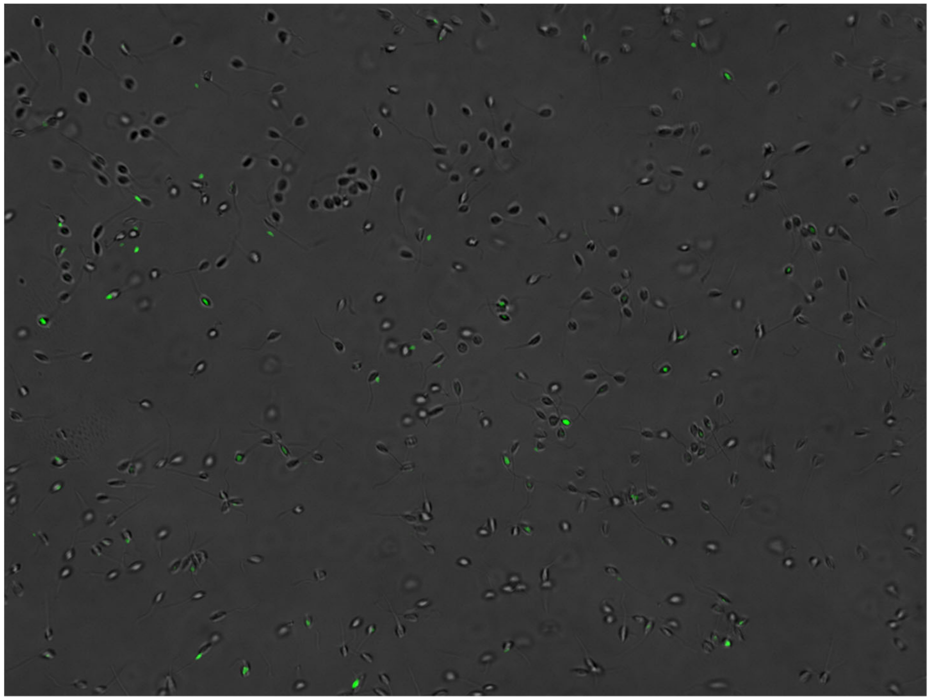

Figure S18. Overlay image of compound **1e** incubation.

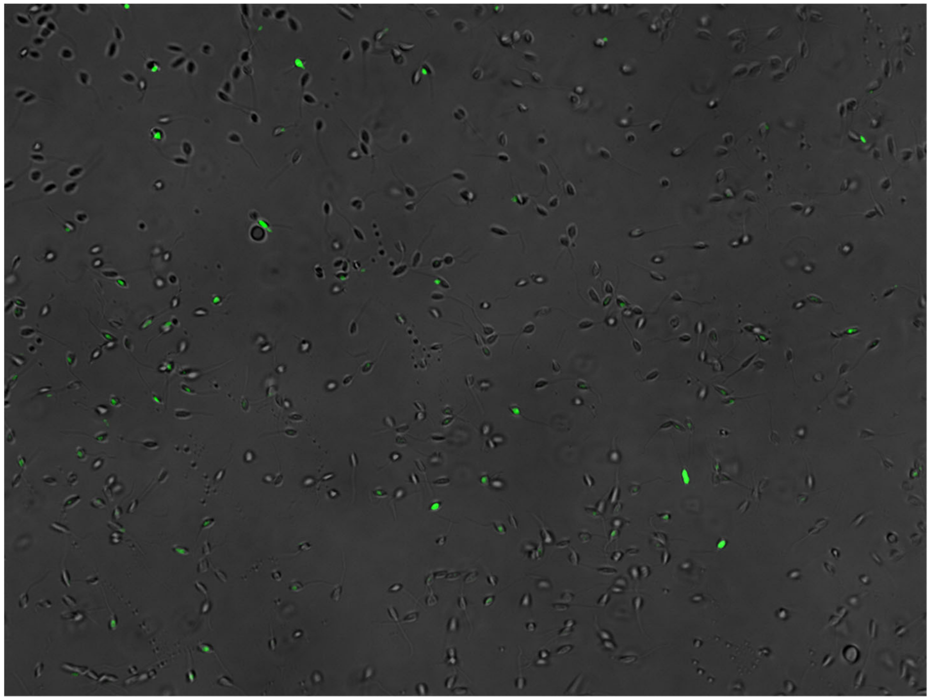
Figure S19. Overlay image of mixed incubation of compounds **1b** + **1e**.

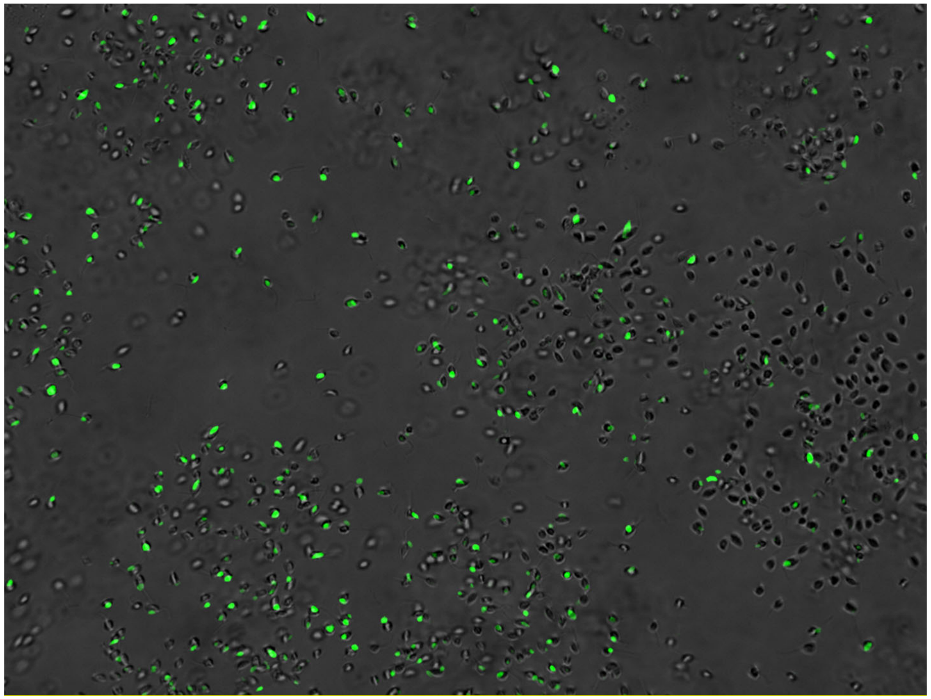

Figure S20. Overlay of mixed incubation of compounds **1c** + **1e**.
